# Genomic Imprinting and Cancer: From Primordial Germ Cells to Somatic Cells

**DOI:** 10.1100/tsw.2006.318

**Published:** 2006-05-26

**Authors:** Adele Murrell

**Affiliations:** Department of Oncology, University of Cambridge, MRC-Hutchison Centre, Hills Road Cambridge CB2 2XZ, UK

**Keywords:** cancer genomic imprinting, loss of imprinting, LOI, Beckwith Wiedemann syndrome, insulin-like growth factor 2, IGF2, Wilms' tumour, epigenetics, stem cells

## Abstract

Imprinted genes are a subset of genes that are expressed from only one of the parental alleles. The majority of imprinted genes have roles in growth regulation and are, therefore, potential oncogenes or tumour suppressors. Cancer is a disease of aberrant cell growth and is characterised by genetic mutations and epigenetic changes such as DNA methylation. The mechanisms whereby imprinting is maintained in somatic cells and then erased and reset in the germline parallels epigenetic changes that cancer cells undergo. This review summarises what we know about imprinting in stem cells and how loss of imprinting may contribute to neoplasia.

## INTRODUCTION AND BROAD OUTLINE

Mammalian development requires both a maternal and a paternal genome[1,2] due to a phenomenon called genomic imprinting in which a subset of genes involved in growth and development are expressed from only one parental allele (reviewed in[3,4]). The imprint is established in the primordial germline (germ stem cells destined to become sperm or ova) and the memory of these parental marks are maintained and read after fertilisation and subsequent development resulting predominantly in monoallelic gene expression. Although the epigenetic imprinting mark is set up in the germline, parent of origin differential expression does not necessarily occur in all cells and in some cases may be tissue and developmental stage specific. This is presumably due to additional acquired chromatin modifications during somatic differentiation and differences with which various transcription factors can "read" the imprints in different tissues.

The nature and mechanism whereby the primary epigenetic mark is acquired in the male and female germline is still not understood despite the fact that imprinted genes have been shown to have allelic differences in DNA methylation and in features of the underlying chromatin such as histone modifications, nucleosomal spacing, and chromatin looping. DNA methylation is an adequate marker to indicate the presence of an imprint even though it may not necessarily be the primary gametic mark. Most imprinted genes have CpG-rich regions where the methylation differs between the parental alleles known as differentially methylated regions (DMRs). Some of these DMRs are acquired in the primordial germline and others are acquired subsequent to fertilisation. The DMRs function as epigenetic response elements and have diverse roles such as imprinting control regions, boundary elements, silencers, and activators[5,6,7,8,9]. Differential histone modifications also occur in imprinted genes at these DMRs[10,11,12] and these may serve as signals that mark parental alleles. There is evidence that a histone modification on H3 Lys9 (H3-K9) could be an imprinting signal that marks imprinted alleles[13,14]. The differences between the parental genomes are then further enhanced in the zygote by the preferential association of heterochromatin-associated protein HP1 to the maternal genome, which is rich in H3-K9 modifications[15,16,17,18]. The primary gametic imprints are inexplicably resistant to genome-wide epigenetic reprogramming events such as global demethylation and *de novo* methylation during early development, and yet they are completely erased prior to establishment of new imprints in the germline[19,20]. Silencing of imprinted genes does not require unique chromatin modifiers that differ from silencing factors in nonimprinted genes and, similarly, expression of imprinted alleles is not reliant on unique transcriptional activation factors. To date, all the biochemical components that have been associated with genomic imprinting have further roles in expression and silencing of nonimprinted genes and are constituents of the cell's natural repertoire of regulatory mechanisms (see Table 2 at end of paper).

The consequence of genomic imprinting is that maternal and paternal genomes of mammals have different effects on embryonic development. These differences are demonstrated by androgenotes and parthenotes. Androgenotes (embryos with an entirely paternal genome) in mice exhibit increased growth of the extraembryonic membranes and die at early midgestation. In humans, an embryo with a paternally derived genome develops trophoblastic cells that can transform into malignant choriocarcinoma (reviewed in [21]). In contrast, parthenotes (embryos with an entirely maternal genome) have retarded development and also die before midgestation[22]. Parent-specific gene expression has been proposed to be the outcome of an evolutionary conflict between the two alleles at a diploid locus of an offspring over how much to demand from parents; thus, the maternally expressed imprinted genes that are growth suppressors while those that are paternally inherited promote growth[23].

As the majority of imprinted genes are involved in growth regulation, they have an inevitable role in neoplasm if they are aberrantly expressed. Loss of imprinting (LOI) results in either biallelic expression due to activation of the silent allele or no expression due to suppression of the active allele. Recent experiments in mice where imprinted genes were demethylated showed that LOI can predispose cells to cellular transformation and tumorigenesis[24], supporting the view that genomic imprinting has a critical tumour-suppressor role. It has been shown that maternally and paternally expressed imprinted genes confer opposite effects on proliferation, cell cycle length, and senescence of embryonic fibroblast cultures[25]. In keeping with the above-mentioned conflict theory, maternally inherited expressed genes $p57^{kip2}$ and *M6P*/*Igf2r* retard proliferation while the paternally expressed growth factor *Igf2* stimulates proliferation.

To systematically review the role of imprinted genes in cancer, we need to consider the developmental windows that are crucial for imprint erasure, establishment, and maintenance and to compare the imprinted gene expression profiles of stem cells, differentiated cells, and cancer cells.

## EPIGENETIC CHARACTERISTICS OF STEM CELLS AND CANCER CELLS

Pluripotential embryonic stem (ES) cells can be derived from the inner cell mass of the blastocyst and can give rise to almost all cell types in the body, differentiating into the multipotential primary embryonic lineages of endoderm, mesoderm, and ectoderm; each capable of producing several cell types of its particular lineage. Pluripotential primordial germ cells (PGCs) originate in the epiblast and migrate to the genital ridge after week 5–6 in humans (day 6.5 in mice), whereafter they are referred to as gonocytes (Fig. 1).

Current speculations are that stem cells may represent the cellular origin of cancer, due to their quiescent existence over long periods of time. Whether cancers develop from stem cells or whether somatic cells are transformed to tumour cells is still under intense debate (reviewed [26,27]). In theory, quiescent stem cells can accumulate mutations during the lifetime of an individual, ultimately giving rise to tumours when they start differentiating. A stem cell origin could explain the delay between an effector stimulus and the cancer phenotype appearing. Cancer stem cells can potentially reactivate the silent germline-specific genes and mediate the malignant phenotype.

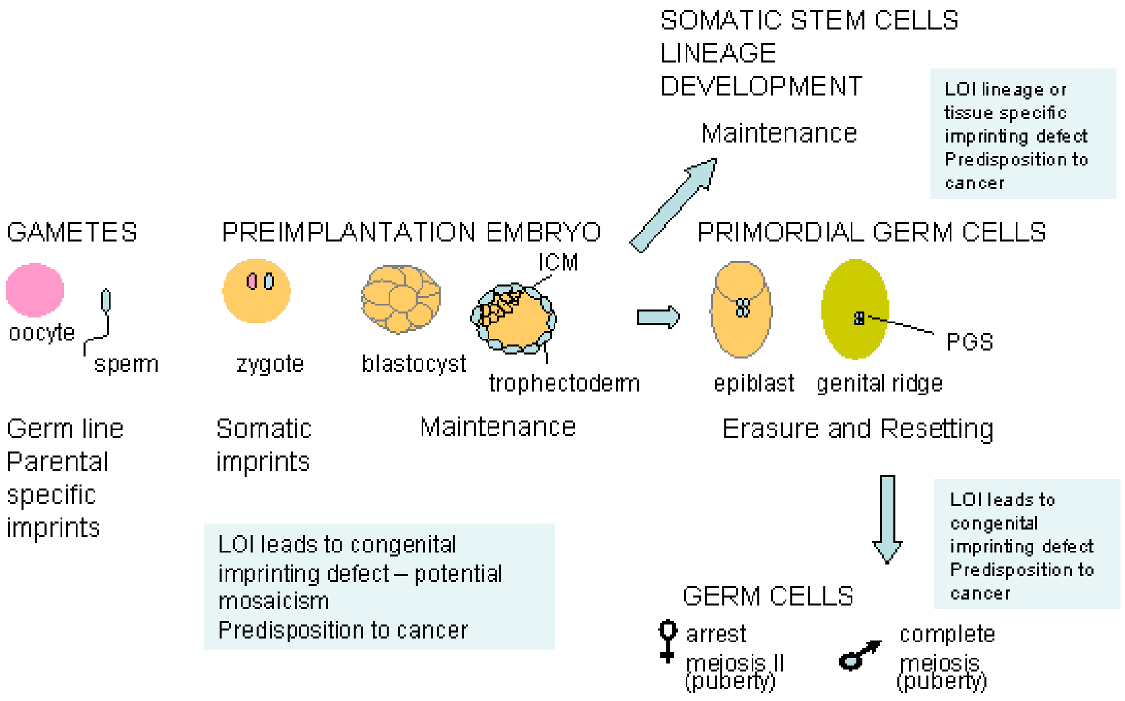

**FIGURE 1.** Ontogeny of imprinting and windows within development in which imprinting can be lost. Oocytes and sperm carry parental-specific germline imprints that were established during germ cell development after PGCs had migrated from the epiblast to the genital ridge. These imprints are stable and resist the genome-wide demethylation and remethylation that occurs after fertilisation during the preimplantation period. Additional somatic imprints are acquired after fertilisation and these, together with the germline imprints, are maintained during lineage development. Events that lead to LOI at the preimplantation stage are failure to establish the somatic imprints and failure to maintain imprinting. This could result in epigenetic mosaicism and, depending on the proportion of cells with LOI, could result in a congenital imprinting defect (such as Prader-Willi/Angelman syndrome; Beckwith Wiedemann syndrome [BWS]) or lineage-restricted LOI with less-severe developmental defects and potential to develop cancers. LOI can also occur after lineage development in somatic stem cells with even further lineage restriction. Global remodelling occurs during germ cell development such that imprints are erased and re-established according to the sex of the embryo so that the gametes will carry new parent-of-origin germline imprints. Mistakes occurring during erasure and establishment of the imprint at this stage will be inherited in the next generation if the gametes with defective imprinting contribute to fertilisation.

A recent model for a polyclonal epigenetic progenitor model of cancer proposes that early epigenetic changes in stem cells give rise to cancer in three steps. The first is an epigenetic alteration of stem/progenitor cells within a given tissue, followed by genetic mutation of tumour suppressor and oncogenes enabling clonal expansion of cells to form benign tumours and primary cancers. The third step is genetic and epigenetic instability that leads to tumour evolution[28].

Shared features between cancer cells and those in the germ cell/gamete/trophoblast differentiation pathways include immortalization (involved in transformation), invasion, induction of meiosis (can lead to aneuploidy), and migration (contributes to metastasis). Germ cells and cancer cells commonly activate growth-promoting genes, silence tumour-suppressor genes, down-regulate the major histocompatibility complex (immune evasion), and undergo epigenetic changes such as global DNA hypomethylation of repeat-rich sequences, hypermethylation of CpG islands associated with gene promoters of specific genes, chromatin alterations, and LOI[29]. In mice, it has been shown that global DNA hypomethylation has a causative role in tumorigenesis[30,31]. Genome-wide hypomethylation has been associated with chromosomal instability and higher mutation rates[32]. Shared characteristics between stem cells and cancer cells do not necessarily indicate that cancers originate from tumour stem cells and it is also likely that genetic alterations in cancer cells activate transformation programmes in which genes normally expressed in stem cells are reactivated. Such a transformation program will also necessitate epigenetic reprogramming. The reversibility of the epigenetic nature of cells is demonstrated by the restoration, albeit infrequent, of pluripotency of a differentiated somatic cell nucleus after it has been transplanted back into an oocyte in a process known as somatic cell transfer (reviewed by [33,34]).

The observation of shared characteristics between germline stem cells and tumour cells plus the detection in tumours of a growing number of cancer/testis (CT) antigens that normally appear to be present only in germ cells and trophoblasts has led to a concept of gametic recapitulation[35]. Thus, during transformation of normal cells to malignancy, normally silent germline expression programmes are reactivated. A new CT gene, *BORIS* (brother of the regulator of imprinted sites), has been demonstrated to reactivate the expression of many CT genes and is proposed to play an antagonistic role to CTCF and to compete for CTCF binding sites[36,37] (see Table 2). The result of BORIS binding to the CTCF recognition sites is to change the methylation status at these sites[37]. BORIS may therefore be a candidate factor for a *trans* effect causing LOI.

The imprinted status and epigenetic profiles of various types of stem cells and the cancers that may develop from these cells are therefore of interest. Human embryonic stem cells derived from *in vitro* fertilised embryos have recently been characterised for expression and methylation status of various paternal and maternally expressed imprinted genes[38]. The paternally expressed *IGF2*, *IPW,* and *KCNQ1OT1* genes and the maternally expressed *H19*, *SLC22A18,* and *NESP55* were shown to be monoallelic in these cells suggesting that monalleleic expression patterns are stable in human stem cells. Methylation patterns of key imprinting control regions at *H19*, *KCNQ1OT1,* and *SNRPN* DMRs were differentially methylated in these cells.

## PRIMORDIAL GERM CELLS, IMPRINTING, AND GONADAL CANCERS

In mice, it has been shown that after erasure of the imprints in primordial germline, the resetting of the imprint (remethylation) of various imprinted genes at different chromosomal loci occurs at different stages of oocyte and spermatocyte development, such that the imprints are acquired asynchronously[39,40,41]. Monoallelic expression and methylation of imprinted genes (*TSSC5*, *H19*, *IGF2*, *SNRPN*) has been observed in human germ cell lines. These germ cell lines were derived from primordial germ cells obtained from the gonadal ridges of 5- to 11-week postfertilization female embryos[42]. In these cell lines, *IGF2* showed partially relaxed imprinting even though the *H19* DMR is normally methylated[42].

Recently, distinct classes of a heterogeneous group of gonadal cancers have been recognised as germ cell tumours based on their expression of specific stem cell proteins and indirectly on their imprinting status. These gonadal cancers actually have imprinting profiles reminiscent of the normal imprinting profile of the proposed germ cell origin of the tumour[43]. Germ cell tumours (types I–V) that arise from PGCs can be present in testes, ovary, sacral region, have recently been extensively reviewed by Oosterhuis and Looijenga[43] and are summarised below in terms of the imprinting profiles shared with their proposed germ cell origins.

Type I and II germ cell tumours are of interest since they are derived from PGCs, which is where the genomic imprint is established. In the majority of these tumours, genomic imprinting is erased or partially erased. Indeed, the imprinting status of these tumours enables the identification of the PGC stage from which the tumours presumably develop. Type I germ cell tumours occur in neonates and children, and typically manifest as teratomas and yolk-sac tumours that occur in the ovaries and testes; sacrococcygeal and retroperitoneal regions; the head and neck; pineal and hypothalamic-hypophyseal region of the brain. Epigenetically, type I germ cell tumours may have either normal biparental genomic imprints or partially erased imprints, indicating that they originated from a more immature PGC than those of type II germ cell tumours in which genomic imprinting is always erased and globally demethylated[43,44,45]. These type II germ cell tumours are also often polyploid (triploid and tetraploid) and reminiscent of chromosomal instability and LOI in mouse models associated with genome-wide hypomethylation[24,30,46,47]. Types III (spermatocytic seminomas) and IV (dermatoid cysts in ovary) germ cell tumours originate in the spermatogonium/spermatocyte and oogonia/oocyte, respectively. The imprinting states of these tumours range from partially-to-completely paternal in the case of spermatocytic seminomas and partially-to-completely maternal in the case of ovarian dermatoid cysts[43,45]. Thus in the case of germline tumours, the imprinting profiles is merely a feature of the PGC. Hydatidiform moles, on the other hand, are clearly an example of imprinted gene expression contributing directly to malignancy. These choriocarcinomas, described below, represent type V germ cell tumours since they originate from fertilised ova.

## HYDATIDIFORM MOLES AND CHORIOCARCINOMAS PROVIDE AN INSIGHT INTO THE MECHANISMS WHEREBY IMPRINTS ARE ESTABLISHED IN THE GERMLINE

Complete hydatidiform moles, which are benign gestational neoplasms with the potential to develop into malignant choriocarcinomas, are an example of the consequence of suppressing all the maternally expressed imprinted genes. Most hydatidiform moles are due to dispermic fertilisation resulting in an androgenetic mole and, hence, have predominant paternal imprints. However, biparental hydatidiform moles also seem to have loss of maternal imprints. The study of hydatidiform moles may hold the key to understanding how the imprint is established in the female germline and how paternally expressed genes in the absence of maternally expressed imprinted genes transform cells to undergo rapid trophoblastic growth. It will be of further interest to determine which of the paternally expressed genes trigger the formation of malignant choriocarcinoma that may arise from molar pregnancies.

In rare instances, families present with recurrent biparental hydatiform moles presumably due to a recessive maternal effect. One such family was thoroughly investigated for methylation of imprinted genes and it was found that the mole tissue had a global absence of methylation on imprinted genes that are normally methylated on the maternal allele[48]. It is thus likely that the genes mutated in these families have a role in establishing the maternal imprint.

*De novo* DNA methyltransferases are the most likely candidates for establishing imprints on maternal oocytes. Indeed, female mice lacking functional *Dnmt3L* or *Dnmt3a* genes also show failure-to-methylate imprinted genes that are usually maternally methylated[49,50,51]. This evidence is in favour of *Dnmt3a* and *Dnmt3L* functioning in the establishment of imprinting in the oocyte, at least, in mouse[49,50,52]. In humans, *Dnmt3L* is not present in oocytes[53]. Moreover, no known *DNMT* gene has been shown to be mutated in families with biparental hydatidiform moles, including the human homologue of an oocyte-specific isoform of the mouse *Dnmt1* gene, *DNMTo*[54]. Hence, the elusive primary imprinting mark may still be a histone modification rather than DNA methylation and, in these families, a histone-modifiying enzyme such as a histone methyltransferase may be implicated. The DNMTS have been shown to interact with histone deacetylase HDAC1 and may, therefore, mediate silencing independently of their DNA methyltransferase activity[52,55]. Changes in DNA methylation influence histone modifications[56,57,58] and conversely, histone modifications can affect DNA methylation[59]. Surprisingly, earlier this year, Murdoch et al identified mutations within the NALP7 gene in familial recurrent biparental hydatidiform moles. NALP7 is involved in inflammatory and apoptotic pathways and there is no obvious functional link between NALP7 and imprint establishment[204].

In contrast to the gonadoblastomas and hydatidiform moles, most tumours show LOI for only a few imprinted genes. Congenital imprinting abnormalities of specific chromosomes that lead to clinical syndromes such as Beckwith Wiedemann syndrome also have a higher instance of childhood cancers and cancer predisposition in adulthood[3,60,61,62,63]. These diseases have varying molecular aetiologies and genotype-phenotype correlations give some clues as to the contribution of LOI to neoplasia and also heritable cancer risk.

## CONGENITAL IMPRINTED SYNDROMES

LOI can be due to genetic mutation or epimutation, and if these occur in the germline or even very early in preimplantation development, they have the potential to underlie hereditary cancer syndromes. Congenital diseases that occur due to disruption of imprinting or dysfunction of one or more imprinted genes include Beckwith Wiedemann, Silver-Russel, Prader-Willi (PWS), Angelman, Transient Neonatal Diabetes Mellitus (TNDM), Albrights hereditary osteodystrophy (AHO), pseudohypothyroidism type 1a (PHP-1a), and PHP-1b (reviewed by [64]). Molecular defects both genetic and epigenetic that give rise to these syndromes, arise at specific imprinted loci in the germline prior to fertilisation or during the peri-implantation development. The perimplantation periods are critical for the incorporation of epigenetic errors due to the enormous amount of reprogramming that occurs during these phases. The erasure and establishment of imprinting of various imprinted genes in the germline has been shown to occur at different stages in germline development[39], suggesting that the imprints are possibly established by different mechanisms at each locus. This asynchrony makes LOI unlikely at all imprinted genes. LOI seems to be an event affecting a single cluster of imprinted genes or even a single imprinted gene. After fertilisation at the two pronuclei stage, the maternal and paternal genomes are each demethylated. The rate of demethylation differs on the maternal and paternal pronuclei with the paternal genome being rapidly demethylated prior to replication and the maternal genome being passively demethylated during replication. Global *de novo* methylation establishes the somatic cell pattern of DNA methylation following implantation[20,65]. Genomic imprints already laid down in the germline are resistant to these global demethylation and remethylation events. In addition to the germline imprints, additional imprints are acquired somatically during postfertilisation demethylation and remethylation. At the mouse *Igf2* locus, it has been shown that CTCF binding at the *H19* ICR influences the methylation of these additional imprints[66,67,68,69,70,71] (Fig. 2).

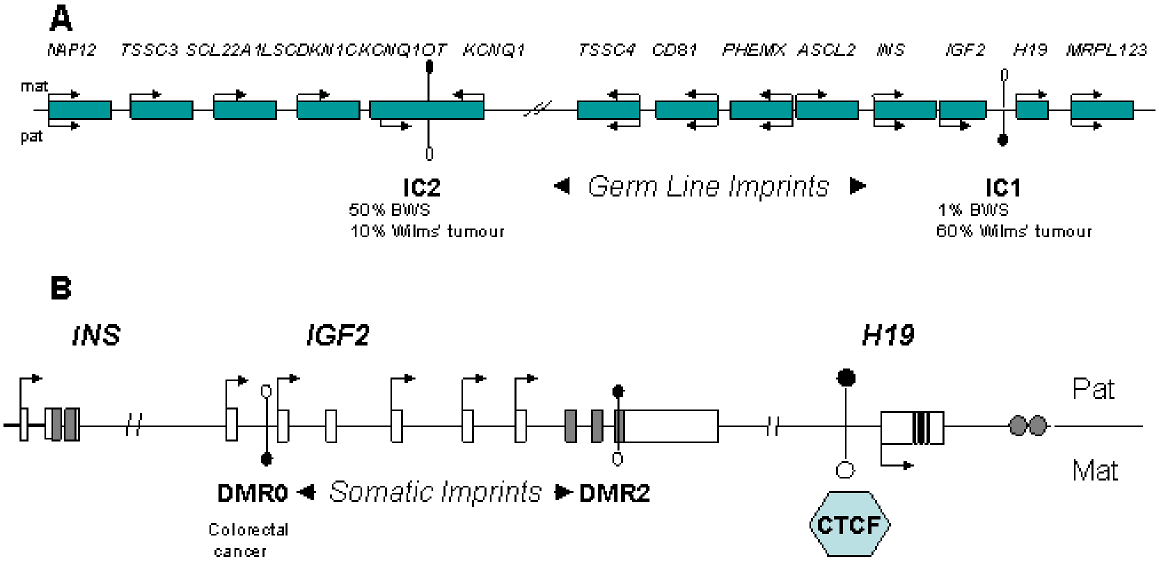

**FIGURE 2.** Germline and somatic imprints that are associated with the cluster of imprinted genes on chromosome 11p15.5. (A) Genes within the cluster at 11p15.5, with arrows depicting direction of transcription for maternal and paternal expression above and below the line. Germline imprints IC1 and IC2 (imprinting control centres 1 and 2) are shown as open and filled lollipops corresponding to unmethylated and methylated regions. IC1 (also sometimes referred to H19 DMR, DMD, and ICR) regulates *H19* and *IGF2* expression, while IC2 (also referred to Lit1 DMR and KvDMR1) regulates *TSSC3*, *SLC22A1LS*, *CDKNIC*, *KCNQ1OT1*, and *KCNQ1*. (B) The IGF2-H19 region showing INS, IGF2, and H19, the germline DMR; IC1 depicted as lollipops as above and the somatic DMRs within IGF2 depicted as slightly smaller lollipops. Methylation at IC1 limits the binding of CTCF to the unmethylated maternal allele and prevents *IGF2* from gaining access to the enhancers (circles) downstream of *H19*. Changes in methylation at germline imprints have been associated with LOI of *IGF2* and other imprinted genes in BWS and childhood tumours such as Wilms' tumour, while in adult cancers, changes in methylation at the somatic DMR0 in IGF2 have been associated with LOI of *IGF2*.

Imprinted genes occur in clusters and share regulatory mechanisms through long-range interactions between regulatory elements. Genetic aetiologies that underlie congenital imprinting syndromes include uniparental disomy (UPD), i.e., the inheritance of a chromosome or chromosome segment from a single parent, other chromosomal abnormalities such as translocations, deletions, or duplications and rarely point mutation. UPD is normally mosaic and arises due to meiotic nondisjunction followed by trisomy rescue and there is evidence that the presence of other chromosomal abnormalities increase the risk of UPD. Somatic recombination during mitosis is another mechanism whereby partial UPD can arise for a chromosomal segment and could be a mechanism for LOI postfertilisation. Partial UPD is quite difficult to detect and the small number of reports in the literature imply that it is underestimated or happens very rarely. Recent data suggest that somatic recombination leading to UPD could be a key event leading to loss of heterozygosity (LOH) without changing copy numbers of chromosomes in basal cell carcinomas[72]. Interestingly, the chromosomal regions involved in this study did not include imprinted genes. The dynamics and frequency with which somatic cell recombination occurs in proliferating and differentiated cells is not known. Chromosome instability and hypomethylation in cancer cell lines have been shown to increase the rate of somatic cell recombination[73].

Some of the syndromes associated with imprinting disorders also have an increased risk to cancer. Imprinting syndromes in which a predisposition to neoplasia has been reported include Prader-Willi Syndrome (PWS) (OMIM #176270), where there is an increased risk for myeloid leukaemia but not other cancers[74]; Albright hereditary osteodystrophy (OMIM #103580 see below for summary) and Beckwith Wiedemann syndrome (OMIM #130650).

## The GNAS Locus: McCune-Albright Syndrome, Albright Hereditary Osteodystrophy, Pseudohypoparathyroidism Type 1A, and Pseudohypoparathyroidism Type 1B

The *GNAS* gene located on chromosome 20q13 has multiple promoters, each generating a different imprinted gene transcript with various functions. These include *NESP55* (neuroendocrine secretory protein 55), *NESP55AS* (antisense *NESP55*), *XL αs* (Golgi-specific $G_s\alpha$ isoform), Exon 1A (untranslated transcript), and $G_s\alpha$ (G protein alpha-subunit). This gene is emerging as a good example of a congenital imprinting defect that can result in various syndromes depending on the inheritance. It is also a potential marker to distinguish between different types of endocrine tumours and contributes to a hereditary predisposition to human growth hormone–secreting pituitary tumours and thyroid tumours. LOI at this locus also occurs in a subset of pituitary adenomas in the absence of congenital syndromes associated with *GNAS*.

*NESP55* is expressed exclusively from the maternal allele and the DMR associated with its promoter is methylated on the paternal allele. It is normally expressed in the chromaffin cells of the adrenal medulla, β pancreatic islet cells, and the pituitary[75]. *NESP55* expression has been shown in a subset of neuroendocrine tumours such as pheochromocytomas, neuroblastomas, pancreatic endocrine tumours, but not in gastrointestinal and pancreatic carcinomas. Thus tumours that express *NESP55* originate from *NESP55* expressing cell lineages, making NESP55 a candidate diagnostic marker for neuroendocrine tumours.

*NESP55AS* and *XLαs* are paternally expressed, as is the untranslated transcript initiating from Exon1A. These promoters are associated with DMRs that are methylated on the maternal allele. LOI of the 1A transcript leads to renal parathyroid hormone resistance PHP-1b. Patients with sporadic PHP-1b have been shown to also have LOI of *NESP55*[76]. Rare familial cases of PHP-1b have been described in which a mutation in a nearby gene *STX16* is associated with LOI at Exon 1A, indicating a long-range interaction between an element within *STX16* and the DMR at Exon 1A in *GNAS*. LOI at Exon 1A occurs in a subset of growth hormone pituitary adenomas[77].

The $G_s\alpha$ promoter is upstream of the first *GNAS* exon and in the majority of tissues the $G_s\alpha$ transcript is biallelically expressed. Tissue-specific imprinting in renal proximal tubules, thyroid, pituitary, and ovary occurs with the maternal allele being expressed. This is not accompanied by methylation changes at the $G_s\alpha$ promoter. Activating missense mutations at the $G_s\alpha$ transcript causes McCune-Albright syndrome (MAS, OMIM #174800). These individuals have a high incidence of various endocrine tumours in addition to multiple congenital defects. Inactivating mutations of the $G_s\alpha$ transcript lead to Albright hereditary osteodystrophy (AHO) and parathyroid, thyroid, and gonadotrophin hormone resistance (PHP-1a) when maternally inherited. The PHP-1a phenotype is due to lack of $G_s\alpha$ expression in the tissues where this transcript is normally imprinted. With paternal transmission of the inactivating mutations, AHO is transmitted without PHP-1a, due to the normal maternal expression of $G_s\alpha$ transcript (reviewed by [78]).

## Beckwith Wiedemann Syndrome

BWS results from mutation and epimutation at the imprinted locus 11p15.5 and is a spectrum of disorders including fetal overgrowth, exomphalos, hemihypertrophy, and predisposition to childhood cancers. The majority of BWS cases are sporadic with a high incidence of UPD and epimutations. Children with BWS have been reported to have a 7.5–9% overall risk for developing embryonal tumours within the first 5–8 years of age (reviewed [3,62,63,79]). The most common tumours include Wilms' tumour and hepatoblastoma, but others including rhabdomyosarcoma, adrenocortical carcinoma, and neuroblastoma have been reported[60,80,81,82,83,84].

Epigenotype/genotype and phenoptype correlations indicate that the risk of neoplasia is increased in cases with UPD. The study of Wilms' tumour has led to some insight on the role of imprinting in neoplasmic formation and these findings are summarised below.

## Wilms' Tumour

Wilms' tumour or nephroblastoma, arises from pluripotent embryonic kidney precursor cells and is the most common paediatric kidney cancer in children[85]. Along with retinoblastoma and neuroblastoma, Wilms' tumour was one of the original tumours to be studied by Knudson during his development of the "two-hit" hypothesis of tumour suppression[86]. The 11p13 locus harbours the *WT1* gene that encodes an embryonic kidney-specific zinc-finger transcription regulator essential for normal renal development. Up to 24 different isoforms of WT protein can be generated. Four major isoforms are generated via combinatorial splicing while the rest are generated from alternative translation initiation sites and RNA editing. Mutations in this gene account for only10–15% of Wilms' tumours.

Some of the earliest studies of epigenetic events were also undertaken in Wilms' tumour[87,88]. The imprinted 11p15.5 domain has a high prevalence of LOH (with significant bias towards allelic loss of maternal alleles) in sporadic tumours and is historically thought to be the locus of a second WT candidate gene. *H19* may in fact be the second Wilms' tumour suppressor gene because of its involvement in the regulation of *IGF2* imprinting. The strongest evidence for this comes from the fact that in genotype-phenotype studies of BWS, changes in the imprinting control region upstream of the *H19* gene (known as IC1 or ICR or *H19* DMR) is more often associated with Wilms' tumour than other subtypes of BWS. The risk for BWS children with epimutation in IC1 or UPD developing Wilms' tumour is 24%, which is higher than the general risk of BWS developing neoplasia (see Fig. 2). Loss of *IGF2* imprinting due to gain of methylation at the IC1 has been detected in up to a third of sporadic Wilms' tumours. Familial cases of BWS with Wilms' tumour have been described in which there is a microdeletion eliminating CTCF binding sites from the IC1 upstream of H19, although there is some controversy as to exactly which CTCF site is required for LOI of *IGF2* and also whether a deletion on its own is enough to cause BWS with Wilms' tumour[89,90,91].

Genomic profiling including screens for both LOH and LOI in Wilms' tumours have shown that LOI occurs more frequently than LOH in early-stage tumours, indicating that LOI is an early event in tumourgenesis[92]. No reports of *IGF2* or indeed any of the genes in the imprinted cluster at 11p15 have been reported to be direct targets for WT1. Interestingly, a recent report has described an antisense *WT1* transcript that is monoalleleically expressed from the paternal allele and, moreover, this transcript shows LOI in Wilms' tumours. A further locus where LOH has been described in Wilms' tumour is 16q which is provocative since this is the location of the *CTCF* gene. Indeed, there is evidence indicating that deletion of *CTCF* leads to changes in methylation at the *IGF2-H19* DMR[93], but the above study suggests LOI precedes LOH of 16q[92].

## POLYMORPHIC IMPRINTING

Polymorphic imprinting refers to inter-individual variations of imprinting. Thus some individuals in a population may show no imprinting for a known imprinted gene, or show imprinting for a gene not normally imprinted in the majority of humans. Polymorphic loss of imprinting has been described for *IGF2*[94], which is normally paternally expressed, and polymorphic gain of imprinting has been reported for *IGF2R* and *WT1,* which are not normally imprinted in humans, but are expressed from the maternal allele in mice[95,96,97,98]. Interestingly, in the case of *IGF2R*, there is still a conserved DMR that displays differential methylation despite biallelic expression[99]. An antisense transcript *Air* regulates imprinting at the *Igf2r* locus in mice[100], but there is no evidence for such a transcript in even in humans samples in which polymorphic imprinting has been identified[101]. Thus, the mechanism whereby *IGF2R* is imprinted differs in mouse and human. Relaxed imprinting of some maternally expressed genes in humans have had the advantage of increasing the expression of tumour-suppressor genes. It is not known under what selective pressure imprinting of the IGF2R was lost at the stage of divergent evolution between primates and other mammals[102]. Polymorphic "gain of imprinting" is likely to decrease the amount of *IGF2R* expression and potentially be a marker for predisposition to cancer. *IGF2R* has been reported to be a tumour suppressor in liver and breast tumours[103,104,105], but although IGF2R expression has been shown to be decreased in tumours, this has been shown to be due to LOH rather than "gain of imprinting"[105]. To date, no individuals have been identified with polymorphic imprinting at *IGF2R* and cancer.

There may be heritable differences between individuals in the fidelity with which allelic DNA methylation differences are established or maintained. Familial studies describing variability between individuals and parent-of-origin DNA methylation differences at specific human Alu elements has recently been reported by Sandovici et al.[106]. In this study, individuals who exhibited high levels of methylation at specific Alu elements came from families in which more than one member also exhibited abnormal patterns of methylation at the differentially methylated regions of the *IGF2/H19* or *IGF2R* loci. Relaxation of imprinting has been demonstrated in a Japanese population where 10.5% of unrelated individuals showed biallelic IGF2 expression[107]. In this same study, it was shown that LOI was confined to IGF2 expression since *SNRPN* imprinting is stringently regulated. Based on these data, LOI of *IGF2* is estimated to occur in 5–10% of the population. LOI of *IGF2* has been associated with an increase risk for colorectal cancer[108].

*SLC22A18 (*SLC22A1L/*IMPT1*/*TSSC5/ORCTL2*) is imprinted in mice and reported to be normally maternally expressed in the embryo. This gene was shown to be biallelic in both Japanese and North American population samples with variable allelic bias towards the maternal allele. In this study, imprinting variability was shown not to be age dependent and the allele bias was stable over time[107]. Differences in allele expression has been shown for nonimprinted genes, suggesting that allelic variation in gene expression may be a common occurrence and yet be an added factor contributing to human variability[109]. Gain of imprinting of *SLC22A18*, with reduction of expression, has been reported in hepatocarcinoma cells[110] and in breast cancer.

What is not known in these studies is whether the polymorphic LOI is present in all tissues and all cells or whether it is tissue specific or even mosaic. Single cell FISH analyses has shown that all combinations of monoallelic and biallelic *Igf2-H19* expression can be found in individual mouse embryonic liver cells, which when analysed by PCR, generally show monoallelic *Igf2* and *H19* expression[111]. It is conceivable that the same mosaicism exists in humans and that in certain individuals, the ratios between cells biallelic and monoallelic for IGF2 can differ so that they appear to be biallelic when they are in fact mosaic. Mosaicism may also explain why the 10% of individuals with LOI at the *IGF2* locus do not present with fetal overgrowth or any features of BWS.

The studies of Sandovici that show familial LOI[106,112] and those of Cui et al.[108,113] that show familial colorectal cancer associated with *IGF2* LOI, suggest an underlying heritable modifier of imprinting. The number of candidates for such a modifier gene is large and includes the all-genes-encoding proteins with a chromatin remodelling and histone modifiers as well as DNA methyltransferases and even components of the folate and homocysteine metabolic pathway.

Epigenetic and genetic factors are likely to interact. Thus, variable DNA methylation can be influenced by sequence variation in *cis* over long distances or in *trans* by proteins that bind to the DNA. Large-scale human epigenome projects will hopefully identify methylation variable positions (MVPs) within the human genome[114]. Quantitative complex trait analyses will need to take variability of DNA methylation patterns and nucleotide variations into account when dissecting the *cis* and *trans* effects of the modifiers.

## MAINTENANCE OF IMPRINTING; LOI AT *IGF2* IN ADULT CANCERS

*IGF2* overexpression plays a pivotal role in tumorigenesis. Aberrant expression of *IGF2* has a role in tumour formation, regardless of imprinting due to its function as a mitogen. In fact, *IGF2* has been reported to be upregulated in cancers without evident loss of imprinting[115]. Although *IGF2* is the most widely reported gene to have LOI in cancer, a growing number of imprinted genes including *ARH1*, *PEG1/MEST, DLK-GTL2* (and others, see Table 1) have been reported to have LOI or/and be aberrantly expressed in various adult cancers.

The stem cell vs. the somatic cell mutation theory is pertinent to the mechanisms whereby imprinting is lost. Thus, depending on whether cancer is of stem cell origin or somatic cell origin will dictate whether observed LOI in cancers is due to a failure to establish the parental imprint or a breakdown in maintenance mechanisms and failure to read the imprint.

LOI in cancer due to failure to establish imprint would suggest a germ cell origin of cancer and be the basis for LOI being a predisposing marker for cancer. LOI of *IGF2* is associated with an increase in progenitor cells in colonic epithelium in humans[46]. In mouse models, it has been shown that LOI of *Igf2* has a causal role in colon cancer[46]. LOI of *IGF2* has been detected in the adjacent normal tissue as well as the tumour cells in colon cancer[108], breast cancer [108], and adenocarcinomas[116]. This could be due to the tumour microenvironment affecting the epigenetic state of progenitor cells or congenital LOI predisposing to cancer.

LOI in the absence of a congenital imprinting syndrome could be because LOI occurred after fertilisation and sometime before or during early lineage differentiation of somatic cells. A small mosaic population of aberrantly imprinted cells could thus arise in any individual. In a stem cell model, LOI could be present in a population of adult stem cells. In this case, the mechanism whereby LOI occurs could be a failure to maintain the imprint and be linked to cell cycle and DNA repair events. Observations that link DNA repair pathways to maintenance of imprinting include colorectal cancer patients with microsatelite instability (MSI) and hypermethylation of the mismatch repair gene *MLH1*[117], as well as patients with MSI and LOI of *IGF2*[118,119]. *Dnmt1* was identified in a screen for mismatch repair genes and *Dnmt1*-deficient ES cells exhibit MSI[120], directly linking maintenance of methylation with DNA repair as suggested previously[121,122,123].

Other than the DNMTs, maintenance of imprinting can depend on the binding of proteins such as CTCF, which protects against methylation[67,69], or the methyl-binding domain proteins (MBDs) which can bind to methylated sequences and recruit chromatin modifying complexes including histone deacetylases[14,124,125]. During DNA replication, several chromatin remodelling factors (histone chaperones, histone modifying enzymes, ATP-dependent remodelling complexes) interact at the replication fork, providing the opportunity to erase DNA methylation or introduce new histone variants. Histones can also be replaced independent of replication. For instance, double-stranded DNA breaks result in phosphorylation of H2A which recruits other histone modifiers and chromatin remodelling complexes to sites of DNA damage (reviewed in [126]).

**TABLE 1**
**Imprinted Genes Involved in Human Cancers**

| Chromosome Location | Gene/Transcript | Expressed Allele* | Protein/RNA | Cancer |
|---|---|---|---|---|
| 1p36 | TP73 (P73) | M | Tumour-related protein | A candidate neuroblastoma tumour suppressor. Deregulated in various tumours, but not yet shown to exhibit LOI[136]. |
| 1p31 | DIRAS3; NOEY2; ARH1 | P | Ras homolog aplysia ras homolog I (ARHI) | Follicular thyroid; carcinogenesis [137,138]; breast and ovarian[206] |
| 6q24 | HYMAI | P | misc RNA | [139] |
| 6q24 | PLAGL1 | P | Zinc finger protein | Fusion gene in many cancers; silenced by LOI in ovarian cancer[140,141] |
| 7q21 | PEG10 | P | Retroviral gag pol homologue | Up-regulated in hepatocarcinomas[142,143]; no description of LOI |
| 7q32 | CPA4 | M | Carboxypeptidase | Monoallelic in prostate cancer[144] |
| | MEST | P | Alpha/beta hydrolase fold family | LOI in lung, colon, and breast cancer[145,146,147,148] |
| 11p15 | H19 | M | misc RNA | Reviewed herein |
| | IGF2 | P | Insulin-like growth factor 2 | Reviewed herein |
| | KCNQ1OT1 | P | Antisense transcript | LOI colorectal cancer[149] |
| | CDKN1C | M | Cyclin-dependent kinase inhibitor | Head and neck cancers; hepatoblastoma; Wilms' tumours biallelic CDKN1C expression and LOH of maternal allele[150,151, 152,153,154,155] |
| | SLC22AA18; (SLC22A1LS, TSSC5/ORCTL2/ IMPT1/BWR1A) | P | Efflux transporter like protein regulates drug sensitivity | Normally only imprinted in embryo. Biallelic expression in adults – imprinted in hepatocarcinoma cells[110] and breast cancer (Gallagher 2006). |
| 11p13 | WT1-Alt; WT1AS | P | Zinc finger protein | Wilms' tumour[156] |
| 11q23 | SDHD | PD P | Succinate dehydrogenase, subunit | Hereditary paragangliomas and phaeochromocytomas[157,158] |
| 14q32 | DLK1 | P | Delta-like 1 homolog | Epigenetic changes occur in various human cancers [159] |

| | | | | |
|---|---|---|---|---|
| | GTL2 | M | misc RNA | [159] |
| 19q13 | IMP01/ITUP1 | P | Imprinted transcript variant1 | Gliomas [160] |
| | PEG3 | P | Zinc finger protein | Candidate tumour-suppressor gene glioma[147,161] |
| 20q11 | NNAT | P | Neuronatin | NNAT indirect – LOH in acute myeloid leukemia and myelodysplastic/myeloproliferative disease[162] |
| 20q13 | L3MBTL | P | Polycomb group | LOH in myeloid malignancy[163] |
| 20q13 | NESP55 | M | Neuroendocrine secretory protein 55 | Described herein[75,164,165] |
| | GNASXL I I | P | arge isoform of GS-a | Described herein |
| | Exon-1A | P | misc RNA | Described herein |
| | GS-alpha | M | Stimulatory G-protein, alpha subunit | Described herein |

* P, Paternal; M, Maternal, PD, provisional data.

Given the belt and braces multilayered mechanism of gene silencing, it is feasible that the imprint can be lost in increments, explaining why in adult cancers, LOI of *IGF2* can be found with various combinations of loss/gain of DNA methylation at different DMRs in the locus and also why, in some cases, LOI is not associated with increased IGF2 expression. Detailed analyses of histone modification, together with DNA methylation status at the DMRs of *IGF2* and neighbouring genes, together with quantitative allele-specific expression levels, are required in order to determine the extent to which the imprint is "lost". Unfortunately, analyses of imprinting in tumour samples is often restricted by the availability of informative polymorphisms, difficulty in determining allele specific methylation profiles, limited amount of material for chromatin analyses, and the need to exclude LOH. Thus, most studies provide data on allele-specific expression of some genes and limited methylation analyses of the two imprinting control regions at 11p15. The two imprinting control regions are IC1 (also described as *H19*DMR/ICR/ DMD), which is normally methylated on the paternal allele and directly regulates *IGF2* expression, and IC2 (described as KvDMR1 and sometimes DMR2), which is normally methylated on the maternal allele and regulates *KCNQ1, CDKN1C, SLC22A1L, ASCL2, PHLDA2*, and *TSSC4* (Fig. 2). These DMRs are methylated in the germline and are regarded as germline DMRs. In mice, it has been shown that *Igf2* is regulated only by the IC1 and does not lose imprinting when IC2 is disrupted[127]. Scelfo et al.[128] demonstrated loss of methylation of IC2 in 30–50% of a variety of adult cancers including liver, breast, cervical, and gastric carcinomas. In this study, other genes at 11p15 were concomitantly hypomethylated, thus indicating loss of methylation under control of the IC2. *H19* imprinting, in keeping with being under a separate imprinting control region, was not affected in these cases. In some cases, LOI of *KCNQOT1* (*LIT1*), which is a direct result of loss of methylation of IC2, has been reported together with biallelic *IGF2* expression[110,129]. The evidence therefore suggests that in human tumour cells, LOI of *IGF2* can occur independently of the *H19* imprinting control centre. This has also been observed in BWS where about 50% of sporadic cases have LOI of *IGF2* independent of methylation status of IC1 and some of these also have loss of methylation at IC2[63,79]. Furthermore, single nucleotide polymorphisms(SNPs) within *IGF2* have been associated with loss of methylation at IC2 in BWS[130]. These results suggest that, at least in some disease states, either IC2 may influence *IGF2* expression or that LOI at *IGF2* and loss of methylation at IC2 can occur through a common mechanism. The somatically acquired DMRs within *IGF2* are of interest in this regard.

In mice, somatic DMRs within *Igf2* directly interact with the germline IC1 at *H19* in a parent-of-origin–specific manner[131]. On the maternal allele, DMR interactions are mediated by CTCF

binding[207]. Human *IGF2* has two DMRs, one that is methylated on the paternal allele (DMR2) and the other that is methylated on the maternal allele (DMR0). In different cancers, LOI of *IGF2* is associated with disruption of different methylation marks, suggesting more than one mechanism whereby imprinting is lost. Thus, in a few cancers, a small number of cases have been shown to have biallelic expression of both *IGF2* and *H19* (e.g., ovarian cancer[132], hepatocellular carcinoma[133]). In others, LOI is confined to *IGF2* only with hypomethylation at *IGF2* DMR0 and no correlation to methylation at the *H19* ICR region[113]. In colorectal cancer and Wilms' tumour, loss of methylation at the DMR0 has been tightly correlated with IGF2 LOI[113,134].

A simplistic explanation is that germline imprints such as IC1 and IC2 and somatic imprints such as *IGF2* DMR0 could have varying susceptibility to being reprogrammed. Thus, germline imprints are generally stable once they have been established while somatic imprints that are acquired later are more easily reprogrammed. Applying this explanation to a stem-cell-origin-of-cancer scenario, we would predict that cancers originating from a germline lineage would have disrupted methylation at the germline DMRS (i.e., IC1 and IC2), while cancers originating from somatic stem cells (adult stem cells, epithelial stem cells) are more likely to have loss of methylation at a somatic DMR (i.e., DMR0 in IGF2). Furthermore, abolishing a germline imprint may affect more than one imprinted gene, while losing a somatic imprint may be localised to a single imprinted gene. Loss of germline imprints could be responsible for early-onset cancers, while loss of somatic imprints may be involved in adult cancers. It is also possible to speculate that depending on the DMR involved, there may be varying responsiveness to therapies involving histone acetylase and DNA methyltransferase inhibitors.

Elegant theories remain to be tested. If DNA damage-repair mechanisms are involved in imprint maintenance, it may be just as easy to erase a germline imprint as a somatic imprint. An isolated study has reported that LOI of *IGF2* is also associated with cell proliferation in adult bone marrow cells and that when lymphocytes are stimulated with phytohaemagglutinin(PHA) *IGF2* expression is up-regulated through LOI and gain of methylation at the H19 ICR[135]. It is not known whether this is an *in vitro* effect, but these experiments indicate how easily even a germline imprint can be reprogrammed on transformation of differentiated cells.

## CONCLUSIONS

Tumours are likely to be induced by a combination of epigenetic defects including histone modifications as well as genomic mutations that result in lack of mismatch repair, global changes in gene expression, and increased chromosomal instability. LOI needs to be considered in the context of these changes in order to establish whether LOI plays a role in the onset or the progression of the tumour.

As with other epigenetic changes in tumour cells, LOI needs to be characterised in terms of the ultimate effects on gene expression levels and how the changes in gene expression levels contribute to cancer progression. Finally, we are now in a position to start high-throughput screening for cancer epigenetic signatures and consideration should be given to the practicalities and clinical value of these assays, and whether imprinted genes are likely to be biomarkers for cancer.

## ACKNOWLEDGEMENTS

Research in my lab is supported by a fellowship from Cancer Research U.K. I thank Eamonn Maher for critical review of the manuscript and helpful comments. I apologise to authors whose work I was unable to discuss owing to space limitations.

**TABLE 2**
**Proteins with Roles in Regulating Genomic Imprinting**

## DNA Methyltransferases

- DNMTs transfer methyl groups to cytosines. They interact with histone deacetylase and may mediate silencing independently of their methyltransferase activity[52,55].
- DNMT1 — Maintenance Dnmt[166]. Dnmt1-/- mice fail to maintain methylation at imprinted genes[24,65,167]. DNMTo is the human homologue of an oocye-specific isoform of mouse *Dnmt1*[54].
- DNMT3A, DNMT3B, and DNMT3L — DNMT3A and B essential for *de novo* methylation[168]. DNMT3a and DNMT3b interact with DNMT3L, which has no catalytic DNA methyltransferase domain. Female mice lacking a functional Dnmt3L or Dnmt3a[49,50] fail to methylate maternally methylated imprinted genes.

## Methyl Binding Domain Proteins

- The MBDs have affinity for methylated DNA and contain transcription repression domains (*TRD*) that can recruit histone deacetylase complexes to silence chromatin (reviewed [125]). A subset of the DNMTs and MBD proteins can form RNA-protein complexes, suggesting that RNA participates in DNA methylation-mediated chromatin control[169].
- MECP1 is a complex containing MBD2 and MBD3/Mi2/NuRD. MBD3L1 also interacts with MBD2 and components of the NuRD complex[170].
- Individual components of Mi2/NuRD: MBD3, Mi2, HDAC1, and HDAC2 are expressed from a very early stage of embryo development and localize with constitutive heterochromatin by the blastula stage[171].
- MECP2 is the founding member of MBDs[125,172]. It interacts with Sin3A and HDACs and is implicated in Rett Syndrome. MECP2 binds to DMRs at imprinted genes such as H19[124], UBE3A[173], and DLX4[174]. MECP2 plays a role in chromatin looping at the DLX4 locus[174].
- A family of Zink finger BTB/POZ domain proteins that bind to methylated DNA and mediate repression include KAISO, ZBTB4, and ZBTB38[175]. ZBTB4 and ZBTB38 bind within H19DMR[175]. KAISO has been shown to interact with CTCF[176].

## DNA Deaminases

AID and APOBEC1 are 5-methylcytosine deaminases that can cause C --> T transition mutations in methylated DNA, which with mismatch repair can lead to demethylation. The mouse *Aid* and *Apobec1* genes are coexpressed with *Nanog* and *Stella* in oocytes, and early periimplantation embryos[177].

## Histone-Modifying Proteins

Imprinted genes have differential histone modifications at their DMRs. The most widely studied are histone acetylation/deacetylation and histone methylation/demethylation. Histone modifications that cross-react with DNA methylation are of interest to the establishment and maintenance of imprinting. A recent review deals with these and their relevance to cancer in more depth[178] .

- Histone Acetyl Transferases (HATs) — e.g., PCAF; p300; CBP act as transcriptional activators and coactivators.
- Histone Deacetylases (HDACs) — e.g., HDAC1, HDAC2 are part of Sin3 NuRD complexes and act as transcriptional corepressors.
- Histone Methyltransferases (HMT) — Arginine HMTs include PRMT4 (CARM1), which methylates arginine residues on H3 and acts as transcriptional coactivator in PCAF, P300, and other complexes.

- Lysine HMTs — SET7/9 methylates H3 lysine 4 and has roles in both transcriptional silencing and activation. Methylation on H3 lysine 4 is usually associated with transcription activation [179,180].
- Suv39H1/2 trimethylates H3 lysine 9 represses transcription as part of E2F1 and E2F4 complexes and also has a role in formation of heterochromatin when associated with HDAC1 and HP1. Loss of Suv39 family of histone methyl transferases impairs mammalian heterochromatin and genome stability[181]. Suv4-20h2 methylates H4 lysine 20 trimethylation[182].
- MLL1 methylates H3 lysine 4 and is a transcriptional activator for proliferation of hematopoiesis[183].
- EZH2 is an HMT that acts on H3 lysine 27 which acts as a signal for transcription repression and maintenance of silencing via polycomb repressive complex PRC1[184]. H3 lysine 27 can also be methylated by G9a[184].
- Histone demethylation — LSD1 (KIAA0601) is an amine oxidase found in association with several repressor complexes that recognise dimethyl lysine 4 on histone H3. RNAi knockdown of LSD results in increase of K4 methylation and transcriptional activation. LSD is also part of a complex containing MLL[185,186].
- PADI4 converts unmethylated or monomethylated arginine to citrulline[187].

## Proteins that Bind to DMRs at Imprinted Genes

- CTCF — Zinc finger DNA binding protein that binds diverse DNA recognition sequences and is methylation sensitive. CTCF has been proven to have boundary element (prevents spreading of heterochromatin) and a chromatin insulator (blocks promoter access to enhancers) function (reviewed [188,189]). Binding of CTCF to the DMRs at H19, in addition to being methylation sensitive, also protects against *de novo* methylation[66,67,68,69]. CTCF binds to other imprinted genes at the DMR region[190,191,192] and also plays a role in secondary chromatin conformation at Igf2-H19.
- BORIS — A CTCF paralogue that is expressed during spermatogenesis at a time when CTCFis switched off. BORIS is reactivated in a variety of cancers and it has been shown that reciprocal binding of CTCF and BORIS to the cancer-testes gene NY-ESO-1 coincides with depression of this gene[36]. *In vitro* ectopic BORIS expression can mediate cancer-testis gene activation by 5 azadC[37].
- LSH — Lymphoid specific helicase member of the SNF2/helicase family of chromatin remodelling proteins[193]. Participates in imprinting control in a locus-specific manner. Lsh-/- mice display global hypomethylation and loss of Cdkn1 imprinting, but not other imprinted genes[194,195].
- Heterochromatin associated protein — Chromodomain protein essential for recognising the methylated Lys9 on histone H3[196,197].

## Polycomb Proteins

- EED — Expressed in human ovarian follicles (primordial to primary and secondary stages), and preimplantation embryos (2–4 cell and blastocysts)[198,199,200]. Required for imprinted X inactivation in mice[199,200] and regulation of some imprinted loci[201].
- EZH2 — Expressed in human ovarian follicles (primordial to primary and secondary stages), mature oocytes (metaphase II), and preimplantation embryos (2–4 cell and blastocysts). Ezh2-Eed Polycomb complex regulates placental imprinting of *Kcnq1* domain[202].
- YY1 — Expressed in human ovarian follicles (primordial to primary and secondary stages), mature oocytes (metaphase II) and preimplantation embryos (2–4 cell and blastocysts) binds to mouse PEG3 in a methylation sensitive manner[203].

## References

1. McGrath, J. and Solter, D. (1984) Completion of mouse embryogenesis requires both the maternal and paternal genomes. *Cell* **37(1),** 179.
2. Barton, S.C., Surani, M.A., and Norris, M.L. (1984) Role of paternal and maternal genomes in mouse development. *Nature* **311(5984),** 374.
3. Reik, W. and Maher, E.R. (1997) Imprinting in clusters: lessons from Beckwith-Wiedemann syndrome. *Trends Genet*. **13(8),** 330.
4. Reik, W., Constancia, M., Dean, W., Davies, K., Bowden, L., Murrell, A., Feil, R., Walter, J., and Kelsey, G. (2000) Igf2 imprinting in development and disease. *Int. J. Dev. Biol.* **44(1),** 145.
5. Bell, A.C. and Felsenfeld, G. (2000) Methylation of a CTCF-dependent boundary controls imprinted expression of the Igf2 gene. *Nature* **405(6785),** 482.
6. Constancia, M., Dean, W., Lopes, S., Moore, T., Kelsey, G., and Reik, W. (2000) Deletion of a silencer element in Igf2 results in loss of imprinting independent of H19. *Nat. Genet.* **26(2),** 203.
7. Hark, A.T., Schoenherr, C.J., Katz, D.J., Ingram, R.S., Levorse, J.M., and Tilghman, S.M. (2000) CTCF mediates methylation-sensitive enhancer-blocking activity at the H19/Igf2 locus. *Nature* **405(6785),** 486.
8. Kanduri, C., Pant, V., Loukinov, D., Pugacheva, E., Qi, C.F., Wolffe, A., Ohlsson, R., and Lobanenkov, V.V. (2000) Functional association of CTCF with the insulator upstream of the H19 gene is parent of origin-specific and methylation-sensitive. *Curr. Biol.* **10(14),** 853.
9. Murrell, A., Heeson, S., Bowden, L., Constancia, M., Dean, W., Kelsey, G., and Reik, W. (2001) An intragenic methylated region in the imprinted Igf2 gene augments transcription. *EMBO Rep*. **2(12),** 1101.
10. Grandjean, V., O'Neill, L., Sado, T., Turner, B., and Ferguson-Smith, A. (2001) Relationship between DNA methylation, histone H4 acetylation and gene expression in the mouse imprinted Igf2-H19 domain. *FEBS Lett*. **488(3),** 165.
11. Lewis, A., Mitsuya, K., Umlauf, D., Smith, P., Dean, W., Walter, J., Higgins, M., Feil, R., and Reik, W. (2004) Imprinting on distal chromosome 7 in the placenta involves repressive histone methylation independent of DNA methylation. *Nat. Genet*. **36(12),** 1291.
12. Umlauf, D., Goto, Y., Cao, R., Cerqueira, F., Wagschal, A., Zhang, Y., and Feil, R. (2004) Imprinting along the Kcnq1 domain on mouse chromosome 7 involves repressive histone methylation and recruitment of Polycomb group complexes. *Nat. Genet*. **36(12),** 1296.
13. Xin, Z., Allis, C.D., and Wagstaff, J. (2001) Parent-specific complementary patterns of histone H3 lysine 9 and H3 lysine 4 methylation at the Prader-Willi syndrome imprinting center. *Am. J. Hum. Genet*. **69(6),** 1389.
14. Fournier, C., Goto, Y., Ballestar, E., Delaval, K., Hever, A.M., Esteller, M., and Feil, R. (2002) Allele-specific histone lysine methylation marks regulatory regions at imprinted mouse genes. *EMBO J.* **21(23),** 6560.
15. Arney, K.L., Bao, S., Bannister, A.J., Kouzarides, T., and Surani, M.A. (2002) Histone methylation defines epigenetic asymmetry in the mouse zygote. *Int. J. Dev. Biol*. **46(3),** 317.
16. Cowell, I.G., Aucott, R., Mahadevaiah, S.K., Burgoyne, P.S., Huskisson, N., Bongiorni, S., Prantera, G., Fanti, L., Pimpinelli, S., Wu, R., Gilbert, D.M., Shi, W., Fundele, R., Morrison, H., Jeppesen, P., and Singh, P.B. (2002) Heterochromatin, HP1 and methylation at lysine 9 of histone H3 in animals. *Chromosoma* **111(1),** 22.
17. Santos, F., Peters, A.H., Otte, A.P., Reik, W., and Dean, W. (2005) Dynamic chromatin modifications characterise the first cell cycle in mouse embryos. *Dev. Biol*. **280**, 225.
18. Surani, M.A. (2001) Reprogramming of genome function through epigenetic inheritance. *Nature* **414(6859),** 122.
19. Reik, W. and Walter, J. (2001) Genomic imprinting: parental influence on the genome. *Nat. Rev. Genet*. **2(1),** 21.
20. Reik, W., Dean, W., and Walter, J. (2001) Epigenetic reprogramming in mammalian development. *Science* **293(5532),** 1089.
21. Li, H.W., Tsao, S.W., and Cheung, A.N. (2002) Current understandings of the molecular genetics of gestational trophoblastic diseases. *Placenta* **23(1),** 20.
22. Sturm, K.S., Flannery, M.L., and Pedersen, R.A. (1994) Abnormal development of embryonic and extraembryonic cell lineages in parthenogenetic mouse embryos. *Dev. Dyn.* **201(1),** 11.
23. Moore, T. and Haig, D. (1991) Genomic imprinting in mammalian development: a parental tug-of-war. *Trends Genet*. **7(2),** 45.
24. Holm, T.M., Jackson-Grusby, L., Brambrink, T., Yamada, Y., Rideout, W.M., 3rd, and Jaenisch, R. (2005) Global loss of imprinting leads to widespread tumorigenesis in adult mice. *Cancer Cell* **8(4),** 275.
25. Hernandez, L., Kozlov, S., Piras, G., and Stewart, C.L. (2003) Paternal and maternal genomes confer opposite effects on proliferation, cell-cycle length, senescence, and tumor formation. *Proc. Natl. Acad. Sci. U. S. A.* **100(23),** 13344.
26. Harris, H. (2005) A long view of fashions in cancer research. *Bioessays* **27(8),** 833.
27. Mikkers, H. and Frisen, J. (2005) Deconstructing stemness. *EMBO J*. **24(15),** 2715.
28. Feinberg, A.P., Ohlsson, R., and Henikoff, S. (2006) The epigenetic progenitor origin of human cancer. *Nat. Rev. Genet*. **7(1),** 21.
29. Feinberg, A.P. and Tycko, B. (2004) The history of cancer epigenetics. *Nat. Rev. Cancer* **4(2),** 143.
30. Gaudet, F., Hodgson, J.G., Eden, A., Jackson-Grusby, L., Dausman, J., Gray, J.W., Leonhardt, H., and Jaenisch, R. (2003) Induction of tumors in mice by genomic hypomethylation. *Science* **300(5618),** 489.

31. Yamada, Y., Jackson-Grusby, L., Linhart, H., Meissner, A., Eden, A., Lin, H., and Jaenisch, R. (2005) Opposing effects of DNA hypomethylation on intestinal and liver carcinogenesis. *Proc. Natl. Acad. Sci. U. S. A.* **102(38),** 13580.
32. Chen, R.Z., Pettersson, U., Beard, C., Jackson-Grusby, L., and Jaenisch, R. (1998) DNA hypomethylation leads to elevated mutation rates. *Nature* **395(6697),** 89.
33. Jaenisch, R., Hochedlinger, K., and Eggan, K. (2005) Nuclear cloning, epigenetic reprogramming and cellular differentiation. *Novartis Found. Symp.* **265**, 107.
34. Latham, K.E. (2005) Early and delayed aspects of nuclear reprogramming during cloning. *Biol. Cell*. **97(2),** 119.
35. Simpson, A.J., Caballero, O.L., Jungbluth, A., Chen, Y.T., and Old, L.J. (2005) Cancer/testis antigens, gametogenesis and cancer. *Nat. Rev. Cancer* **5(8),** 615.
36. Hong, J.A., Kang, Y., Abdullaev, Z., Flanagan, P.T., Pack, S.D., Fischette, M.R., Adnani, M.T., Loukinov, D.I., Vatolin, S., Risinger, J.I., Custer, M., Chen, G.A., Zhao, M., Nguyen, D.M., Barrett, J.C., Lobanenkov, V.V., and Schrump, D.S. (2005) Reciprocal binding of CTCF and BORIS to the NY-ESO-1 promoter coincides with derepression of this cancer-testis gene in lung cancer cells. *Cancer Res*. **65(17),** 7763.
37. Vatolin, S., Abdullaev, Z., Pack, S.D., Flanagan, P.T., Custer, M., Loukinov, D.I., Pugacheva, E., Hong, J.A., Morse, H., 3rd, Schrump, D.S., Risinger, J.I., Barrett, J.C., and Lobanenkov, V.V. (2005) Conditional expression of the CTCF-paralogous transcriptional factor BORIS in normal cells results in demethylation and derepression of MAGE-A1 and reactivation of other cancer-testis genes. *Cancer Res*. **65(17),** 7751.
38. Rugg-Gunn, P.J., Ferguson-Smith, A.C., and Pedersen, R.A. (2005) Epigenetic status of human embryonic stem cells. *Nat. Genet*. **37(6),** 585.
39. Hajkova, P., Erhardt, S., Lane, N., Haaf, T., El-Maarri, O., Reik, W., Walter, J., and Surani, M.A. (2002) Epigenetic reprogramming in mouse primordial germ cells. Mech*. Dev.* **117(1–2),** 15.
40. Lucifero, D., Mann, M.R., Bartolomei, M.S., and Trasler, J.M. (2004) Gene-specific timing and epigenetic memory in oocyte imprinting. *Hum. Mol. Genet*. **13(8),** 839.
41. Davis, T.L., Yang, G.J., McCarrey, J.R., and Bartolomei, M.S. (2000) The H19 methylation imprint is erased and re-established differentially on the parental alleles during male germ cell development. *Hum. Mol. Genet*. **9(19),** 2885.
42. Onyango, P., Jiang, S., Uejima, H., Shamblott, M.J., Gearhart, J.D., Cui, H., and Feinberg, A.P. (2002) Monoallelic expression and methylation of imprinted genes in human and mouse embryonic germ cell lineages. *Proc. Natl. Acad. Sci. U. S. A.* **99(16),** 10599.
43. Oosterhuis, J.W. and Looijenga, L.H. (2005) Testicular germ-cell tumours in a broader perspective. *Nat. Rev. Cancer* **5(3),** 210.
44. van Gurp, R.J., Oosterhuis, J.W., Kalscheuer, V., Mariman, E.C., and Looijenga, L.H. (1994) Biallelic expression of the H19 and IGF2 genes in human testicular germ cell tumors. *J. Natl. Cancer Inst*. **86(14),** 1070.
45. Verkerk, A.J., Ariel, I., Dekker, M.C., Schneider, T., van Gurp, R.J., de Groot, N., Gillis, A.J., Oosterhuis, J.W., Hochberg, A.A., and Looijenga, L.H. (1997) Unique expression patterns of H19 in human testicular cancers of different etiology. *Oncogene* **14(1),** 95.
46. Sakatani, T., Kaneda, A., Iacobuzio-Donahue, C.A., Carter, M.G., de Boom Witzel, S., Okano, H., Ko, M.S., Ohlsson, R., Longo, D.L., and Feinberg, A.P. (2005) Loss of imprinting of Igf2 alters intestinal maturation and tumorigenesis in mice. *Science* **307(5717),** 1976.
47. Eden, A., Gaudet, F., Waghmare, A., and Jaenisch, R. (2003) Chromosomal instability and tumors promoted by DNA hypomethylation. *Science* **300(5618),** 455.
48. Judson, H., Hayward, B.E., Sheridan, E., and Bonthron, D.T. (2002) A global disorder of imprinting in the human female germline. *Nature* **416(6880),** 539.
49. Bourc'his, D., Xu, G.L., Lin, C.S., Bollman, B., and Bestor, T.H. (2001) Dnmt3L and the establishment of maternal genomic imprints. *Science* **294(5551),** 2536.
50. Hata, K., Okano, M., Lei, H., and Li, E. (2002) Dnmt3L cooperates with the Dnmt3 family of de novo DNA methyltransferases to establish maternal imprints in mice. *Development* **129(8),** 1983.
51. Kaneda, M., Okano, M., Hata, K., Sado, T., Tsujimoto, N., Li, E., and Sasaki, H. (2004) Essential role for de novo DNA methyltransferase Dnmt3a in paternal and maternal imprinting. *Nature* **429(6994),** 900.
52. Deplus, R., Brenner, C., Burgers, W.A., Putmans, P., Kouzarides, T., de Launoit, Y., and Fuks, F. (2002) Dnmt3L is a transcriptional repressor that recruits histone deacetylase. *Nucleic Acids Res*. **30(17),** 3831.
53. Huntriss, J., Hinkins, M., Oliver, B., Harris, S.E., Beazley, J.C., Rutherford, A.J., Gosden, R.G., Lanzendorf, S.E., and Picton, H.M. (2004) Expression of mRNAs for DNA methyltransferases and methyl-CpG-binding proteins in the human female germline, preimplantation embryos, and embryonic stem cells. *Mol. Reprod. Dev*. **67(3),** 323.
54. Hayward, B.E., De Vos, M., Judson, H., Hodge, D., Huntriss, J., Picton, H.M., Sheridan, E., and Bonthron, D.T. (2003) Lack of involvement of known DNA methyltransferases in familial hydatidiform mole implies the involvement of other factors in establishment of imprinting in the human female germline. *BMC Genet*. **4,** 2.
55. Aapola, U., Liiv, I., and Peterson, P. (2002) Imprinting regulator DNMT3L is a transcriptional repressor associated with histone deacetylase activity. *Nucleic Acids Res*. **30(16),** 3602.
56. Fuks, F., Hurd, P.J., Wolf, D., Nan, X., Bird, A.P., and Kouzarides, T. (2003) The methyl-CpG-binding protein MeCP2 links DNA methylation to histone methylation. *J. Biol. Chem*. **278(6),** 4035.
57. Bachman, K.E., Park, B.H., Rhee, I., Rajagopalan, H., Herman, J.G., Baylin, S.B., Kinzler, K.W., and Vogelstein, B.

(2003) Histone modifications and silencing prior to DNA methylation of a tumor suppressor gene. *Cancer Cell* **3(1),** 89.
58. Espada, J., Ballestar, E., Fraga, M.F., Villar-Garea, A., Juarranz, A., Stockert, J.C., Robertson, K.D., Fuks, F., and Esteller, M. (2004) Human DNA methyltransferase 1 is required for maintenance of the histone H3 modification pattern. *J. Biol. Chem*. **279(35),** 37175.
59. Fraga, M.F., Ballestar, E., Villar-Garea, A., Boix-Chornet, M., Espada, J., Schotta, G., Bonaldi, T., Haydon, C., Ropero, S., Petrie, K., Iyer, N.G., Perez-Rosado, A., Calvo, E., Lopez, J.A., Cano, A., Calasanz, M.J., Colomer, D., Piris, M.A., Ahn, N., Imhof, A., Caldas, C., Jenuwein, T., and Esteller, M. (2005) Loss of acetylation at Lys16 and trimethylation at Lys20 of histone H4 is a common hallmark of human cancer. *Nat. Genet.* **37(4),** 391.
60. Fukuzawa, R., Hata, J., Hayashi, Y., Ikeda, H., and Reeve, A.E. (2003) Beckwith-Wiedemann syndrome-associated hepatoblastoma: wnt signal activation occurs later in tumorigenesis in patients with 11p15.5 uniparental disomy. *Pediatr. Dev. Pathol*. **6(4),** 299.
61. Kerr, N.J., Fukuzawa, R., Reeve, A.E., and Sullivan, M.J. (2002) Beckwith-Wiedemann syndrome, pancreatoblastoma, and the wnt signaling pathway. *Am. J. Pathol*. **160(4),** 1541.
62. Maher, E.R. and Reik, W. (2000) Beckwith-Wiedemann syndrome: imprinting in clusters revisited. *J. Clin. Invest*. **105(3),** 247.
63. Cooper, W.N., Luharia, A., Evans, G.A., Raza, H., Haire, A.C., Grundy, R., Bowdin, S.C., Riccio, A., Sebastio, G., Bliek, J., Schofield, P.N., Reik, W., Macdonald, F., and Maher, E.R. (2005) Molecular subtypes and phenotypic expression of Beckwith-Wiedemann syndrome. *Eur. J. Hum. Genet*. **13(9),** 1025.
64. Robertson, K.D. (2005) DNA methylation and human disease. *Nat. Rev. Genet*. **6(8),** 597.
65. Li, E., Beard, C., and Jaenisch, R. (1993) Role for DNA methylation in genomic imprinting. *Nature* **366(6453),** 362.
66. Fedoriw, A.M., Stein, P., Svoboda, P., Schultz, R.M., and Bartolomei, M.S. (2004) Transgenic RNAi reveals essential function for CTCF in H19 gene imprinting. *Science* **303(5655),** 238.
67. Lewis, A. and Murrell, A. (2004) Genomic imprinting: CTCF protects the boundaries. *Curr. Biol*. **14(7),** R284.
68. Pant, V., Kurukuti, S., Pugacheva, E., Shamsuddin, S., Mariano, P., Renkawitz, R., Klenova, E., Lobanenkov, V., and Ohlsson, R. (2004) Mutation of a single CTCF target site within the H19 imprinting control region leads to loss of Igf2 imprinting and complex patterns of de novo methylation upon maternal inheritance. *Mol. Cell. Biol*. **24(8),** 3497.
69. Schoenherr, C.J., Levorse, J.M., and Tilghman, S.M. (2003) CTCF maintains differential methylation at the Igf2/H19 locus. *Nat. Genet*. **33(1),** 66.
70. Szabo, P.E., Tang, S.H., Silva, F.J., Tsark, W.M., and Mann, J.R. (2004) Role of CTCF binding sites in the Igf2/H19 imprinting control region. *Mol. Cell. Biol*. **24(11),** 4791.
71. Lopes, S., Lewis, A., Hajkova, P., Dean, W., Oswald, J., Forne, T., Murrell, A., Constancia, M., Bartolomei, M., Walter, J., and Reik, W. (2003) Epigenetic modifications in an imprinting cluster are controlled by a hierarchy of DMRs suggesting long-range chromatin interactions. *Hum. Mol. Genet*. **12(3),** 295.
72. Teh, M.T., Blaydon, D., Chaplin, T., Foot, N.J., Skoulakis, S., Raghavan, M., Harwood, C.A., Proby, C.M., Philpott, M.P., Young, B.D., and Kelsell, D.P. (2005) Genomewide single nucleotide polymorphism microarray mapping in basal cell carcinomas unveils uniparental disomy as a key somatic event. *Cancer Res.* **65(19),** 8597.
73. Ciotta, C., Ceccotti, S., Aquilina, G., Humbert, O., Palombo, F., Jiricny, J., and Bignami, M. (1998) Increased somatic recombination in methylation tolerant human cells with defective DNA mismatch repair. *J. Mol. Biol*. **276(4),** 705.
74. Davies, H.D., Leusink, G.L., McConnell, A., Deyell, M., Cassidy, S.B., Fick, G.H., and Coppes, M.J. (2003) Myeloid leukemia in Prader-Willi syndrome. *J. Pediatr*. **142(2),** 174.
75. Jakobsen, A.M., Ahlman, H., Kolby, L., Abrahamsson, J., Fischer-Colbrie, R., and Nilsson, O. (2003) NESP55, a novel chromogranin-like peptide, is expressed in endocrine tumours of the pancreas and adrenal medulla but not in ileal carcinoids. *Br. J. Cancer* **88(11),** 1746.
76. Bastepe, M., Frohlich, L.F., Linglart, A., Abu-Zahra, H.S., Tojo, K., Ward, L.M., and Juppner, H. (2005) Deletion of the NESP55 differentially methylated region causes loss of maternal GNAS imprints and pseudohypoparathyroidism type Ib. *Nat. Genet*. **37(1),** 25.
77. Hayward, B.E., Barlier, A., Korbonits, M., Grossman, A.B., Jacquet, P., Enjalbert, A., and Bonthron, D.T. (2001) Imprinting of the G(s)alpha gene GNAS1 in the pathogenesis of acromegaly. *J. Clin. Invest*. **107(6),** R31.
78. Weinstein, L.S., Liu, J., Sakamoto, A., Xie, T., and Chen, M. (2004) Minireview: GNAS: normal and abnormal functions. *Endocrinology* **145(12),** 5459.
79. Weksberg, R., Shuman, C., and Smith, A.C. (2005) Beckwith-Wiedemann syndrome. *Am. J. Med. Genet. C Semin. Med. Genet*. **137(1),** 12.
80. Thornburg, C.D., Shulkin, B.L., Castle, V.P., and McAllister-Lucas, L.M. (2003) Thoracic neural crest tumors in Beckwith-Wiedemann syndrome. *Med. Pediatr. Oncol*. **41(5),** 468.
81. Garcia Penalver, C., Lopez Vazquez, F., Gomez Fraile, A., and Aransay Bramtot, A. (2003) [Nephroblastomatosis: which therapeutic approach should be used? Report of 2 cases]. *Actas Urol. Esp*. **27(10),** 809.
82. Hertel, N.T., Carlsen, N., Kerndrup, G., Pedersen, I.L., Clausen, N., Hahnemann, J.M., and Jacobsen, B.B. (2003) Late relapse of adrenocortical carcinoma in Beckwith-Wiedemann syndrome. Clinical, endocrinological and genetic aspects. *Acta Paediatr*. **92(4),** 439.
83. Pelizzo, G., Conoscenti, G., Kalache, K.D., Vesce, F., Guerrini, P., and Cavazzini, L. (2003) Antenatal manifestation of congenital pancreatoblastoma in a fetus with Beckwith-Wiedemann syndrome. *Prenat. Diagn*. **23(4),** 292.

84. Davies, S.M. and Ross, J.A. (2003) Childhood cancer etiology: recent reports. *Med. Pediatr. Oncol.* **40(1),** 35.
85. Rivera, M.N. and Haber, D.A. (2005) Wilms' tumour: connecting tumorigenesis and organ development in the kidney. *Nat. Rev. Cancer* **5(9),** 699.
86. Knudson, A.G. (1996) Hereditary cancer: two hits revisited. *J. Cancer Res. Clin. Oncol*. **122(3),** 135.
87. Ogawa, O., Eccles, M.R., Szeto, J., McNoe, L.A., Yun, K., Maw, M.A., Smith, P.J., and Reeve, A.E. (1993) Relaxation of insulin-like growth factor II gene imprinting implicated in Wilms' tumour. *Nature* **362(6422),** 749.
88. Rainier, S., Johnson, L.A., Dobry, C.J., Ping, A.J., Grundy, P.E., and Feinberg, A.P. (1993) Relaxation of imprinted genes in human cancer. *Nature* **362(6422),** 747.
89. Prawitt, D. and Riccio, A. (2005) Microdeletion and IGF2 loss of imprinting in a cascade causing Beckwith-Wiedemann syndrome with Wilms' tumor. *Nat. Genet*. **8,** 785.
90. Sparago, A., Cerrato, F., Vernucci, M., Ferrero, G.B., Silengo, M.C., and Riccio, A. (2004) Microdeletions in the human H19 DMR result in loss of IGF2 imprinting and Beckwith-Wiedemann syndrome. *Nat. Genet*. **36(9),** 958.
91. Prawitt, D., Enklaar, T., Gartner-Rupprecht, B., Spangenberg, C., Oswald, M., Lausch, E., Schmidtke, P., Reutzel, D., Fees, S., Lucito, R., Korzon, M., Brozek, I., Limon, J., Housman, D.E., Pelletier, J., and Zabel, B. (2005) Microdeletion of target sites for insulator protein CTCF in a chromosome 11p15 imprinting center in Beckwith-Wiedemann syndrome and Wilms' tumor. *Proc. Natl. Acad. Sci. U. S. A.* **102(11),** 4085.
92. Yuan, E., Li, C.M., Yamashiro, D.J., Kandel, J., Thaker, H., Murty, V.V., and Tycko, B. (2005) Genomic profiling maps loss of heterozygosity and defines the timing and stage dependence of epigenetic and genetic events in Wilms' tumors. *Mol. Cancer Res*. **3(9),** 493.
93. Mummert, S.K., Lobanenkov, V.A., and Feinberg, A.P. (2005) Association of chromosome arm 16q loss with loss of imprinting of insulin-like growth factor-II in Wilms tumor. *Genes Chromosomes Cancer* **43(2),** 155.
94. Polychronakos, C., Giannoukakis, N., and Deal, C.L. (1995) Imprinting of IGF2, insulin-dependent diabetes, immune function, and apoptosis: a hypothesis. *Dev. Genet*. **17(3),** 253.
95. Kalscheuer, V.M., Mariman, E.C., Schepens, M.T., Rehder, H., and Ropers, H.H. (1993) The insulin-like growth factor type-2 receptor gene is imprinted in the mouse but not in humans. *Nat. Genet*. **5(1),** 74.
96. Jinno, Y., Yun, K., Nishiwaki, K., Kubota, T., Ogawa, O., Reeve, A.E., and Niikawa, N. (1994) Mosaic and polymorphic imprinting of the WT1 gene in humans. *Nat. Genet*. **6(3),** 305.
97. Ogawa, O., McNoe, L.A., Eccles, M.R., Morison, I.M., and Reeve, A.E. (1993) Human insulin-like growth factor type I and type II receptors are not imprinted. *Hum. Mol. Genet.* **2(12),** 2163.
98. Xu, Y., Goodyer, C.G., Deal, C., and Polychronakos, C. (1993) Functional polymorphism in the parental imprinting of the human IGF2R gene. *Biochem. Biophys. Res. Commun*. **197(2),** 747.
99. Smrzka, O.W., Fae, I., Stoger, R., Kurzbauer, R., Fischer, G.F., Henn, T., Weith, A., and Barlow, D.P. (1995) Conservation of a maternal-specific methylation signal at the human IGF2R locus. *Hum. Mol. Genet*. **4(10),** 1945.
100. Wutz, A., Smrzka, O.W., Schweifer, N., Schellander, K., Wagner, E.F., and Barlow, D.P. (1997) Imprinted expression of the Igf2r gene depends on an intronic CpG island. *Nature* **389(6652),** 745.
101. Oudejans, C.B., Westerman, B., Wouters, D., Gooyer, S., Leegwater, P.A., van Wijk, I.J., and Sleutels, F. (2001) Allelic IGF2R repression does not correlate with expression of antisense RNA in human extraembryonic tissues. *Genomics* **73(3),** 331.
102. Killian, J.K., Nolan, C.M., Wylie, A.A., Li, T., Vu, T.H., Hoffman, A.R., and Jirtle, R.L. (2001) Divergent evolution in M6P/IGF2R imprinting from the Jurassic to the Quaternary. *Hum. Mol. Genet*. **10(17),** 1721.
103. Hankins, G.R., De Souza, A.T., Bentley, R.C., Patel, M.R., Marks, J.R., Iglehart, J.D., and Jirtle, R.L. (1996) M6P/IGF2 receptor: a candidate breast tumor suppressor gene. *Oncogene* **12(9),** 2003.
104. De Souza, A.T., Yamada, T., Mills, J.J., and Jirtle, R.L. (1997) Imprinted genes in liver carcinogenesis. *FASEB J.* **11(1),** 60.
105. Yamada, T., De Souza, A.T., Finkelstein, S., and Jirtle, R.L. (1997) Loss of the gene encoding mannose 6-phosphate/insulin-like growth factor II receptor is an early event in liver carcinogenesis. *Proc. Natl. Acad. Sci. U. S. A*. **94(19),** 10351.
106. Sandovici, I., Kassovska-Bratinova, S., Loredo-Osti, J.C., Leppert, M., Suarez, A., Stewart, R., Bautista, F.D., Schiraldi, M., and Sapienza, C. (2005) Interindividual variability and parent of origin DNA methylation differences at specific human Alu elements. *Hum. Mol. Genet*. **14(15),** 2135.
107. Sakatani, T., Wei, M., Katoh, M., Okita, C., Wada, D., Mitsuya, K., Meguro, M., Ikeguchi, M., Ito, H., Tycko, B., and Oshimura, M. (2001) Epigenetic heterogeneity at imprinted loci in normal populations. *Biochem. Biophys. Res. Commun*. **283(5),** 1124.
108. Cui, H., Cruz-Correa, M., Giardiello, F.M., Hutcheon, D.F., Kafonek, D.R., Brandenburg, S., Wu, Y., He, X., Powe, N.R., and Feinberg, A.P. (2003) Loss of IGF2 imprinting: a potential marker of colorectal cancer risk. *Science* **299(5613),** 1753.
109. Lo, H.S., Wang, Z., Hu, Y., Yang, H.H., Gere, S., Buetow, K.H., and Lee, M.P. (2003) Allelic variation in gene expression is common in the human genome. *Genome Res*. **13(8),** 1855.
110. Schwienbacher, C., Gramantieri, L., Scelfo, R., Veronese, A., Calin, G.A., Bolondi, L., Croce, C.M., Barbanti-Brodano, G., and Negrini, M. (2000) Gain of imprinting at chromosome 11p15: a pathogenetic mechanism identified in human hepatocarcinomas. *Proc. Natl. Acad. Sci. U. S. A.* **97(10),** 5445.
111. Jouvenot, Y., Poirier, F., Jami, J., and Paldi, A. (1999) Biallelic transcription of Igf2 and H19 in individual cells suggests a post-transcriptional contribution to genomic imprinting. *Curr. Biol*. **9(20),** 1199.

112. Sandovici, I., Leppert, M., Hawk, P.R., Suarez, A., Linares, Y., and Sapienza, C. (2003) Familial aggregation of abnormal methylation of parental alleles at the IGF2/H19 and IGF2R differentially methylated regions. *Hum. Mol. Genet*. **12(13),** 1569.
113. Cui, H., Onyango, P., Brandenburg, S., Wu, Y., Hsieh, C.L., and Feinberg, A.P. (2002) Loss of imprinting in colorectal cancer linked to hypomethylation of H19 and IGF2. *Cancer Res*. **62(22),** 6442.
114. Murrell, A., Rakyan, V.K., and Beck, S. (2005) From genome to epigenome. *Hum. Mol. Genet.* **14(Spec No 1),** R3.
115. Wang, W.H., Duan, J.X., Vu, T.H., and Hoffman, A.R. (1996) Increased expression of the insulin-like growth factor-II gene in Wilms' tumor is not dependent on loss of genomic imprinting or loss of heterozygosity. *J. Biol. Chem*. **271(44),** 27863.
116. Jarrard, D.F., Bussemakers, M.J., Bova, G.S., and Isaacs, W.B. (1995) Regional loss of imprinting of the insulin-like growth factor II gene occurs in human prostate tissues. *Clin. Cancer Res.* **1(12),** 1471.
117. Esteller, M., Levine, R., Baylin, S.B., Ellenson, L.H., and Herman, J.G. (1998) MLH1 promoter hypermethylation is associated with the microsatellite instability phenotype in sporadic endometrial carcinomas. *Oncogene* **17(18),** 2413.
118. Nakagawa, H., Chadwick, R.B., Peltomaki, P., Plass, C., Nakamura, Y., and de La Chapelle, A. (2001) Loss of imprinting of the insulin-like growth factor II gene occurs by biallelic methylation in a core region of H19-associated CTCF-binding sites in colorectal cancer. *Proc. Natl. Acad. Sci. U. S. A.* **98(2),** 591.
119. Cui, H., Horon, I.L., Ohlsson, R., Hamilton, S.R., and Feinberg, A.P. (1998) Loss of imprinting in normal tissue of colorectal cancer patients with microsatellite instability. *Nat. Med*. **4(11),** 1276.
120. Guo, G., Wang, W., and Bradley, A. (2004) Mismatch repair genes identified using genetic screens in Blm-deficient embryonic stem cells. *Nature* **429(6994),** 891.
121. Trinh, B.N., Long, T.I., Nickel, A.E., Shibata, D., and Laird, P.W. (2002) DNA methyltransferase deficiency modifies cancer susceptibility in mice lacking DNA mismatch repair. *Mol. Cell. Biol*. **22(9),** 2906.
122. Kim, M., Trinh, B.N., Long, T.I., Oghamian, S., and Laird, P.W. (2004) Dnmt1 deficiency leads to enhanced microsatellite instability in mouse embryonic stem cells. *Nucleic Acids Res*. **32(19),** 5742.
123. Wang, K.Y. and James Shen, C.K. (2004) DNA methyltransferase Dnmt1 and mismatch repair. *Oncogene* **23(47),** 7898.
124. Drewell, R.A., Goddard, C.J., Thomas, J.O., and Surani, M.A. (2002) Methylation-dependent silencing at the H19 imprinting control region by MeCP2. *Nucleic Acids Res*. **30(5),** 1139.
125. Wade, P.A. (2001) Methyl CpG-binding proteins and transcriptional repression. *Bioessays* **23(12),** 1131.
126. van Attikum, H. and Gasser, S.M. (2005) The histone code at DNA breaks: a guide to repair? *Nat. Rev. Mol. Cell Biol*. **6(10),** 757.
127. Fitzpatrick, G.V., Soloway, P.D., and Higgins, M. (2002) Regional loss of imprinting and growth deficiency in mice with a targeted deletion of KvDMR1. *Nat. Genet*. **32,** 426.
128. Scelfo, R.A., Schwienbacher, C., Veronese, A., Gramantieri, L., Bolondi, L., Querzoli, P., Nenci, I., Calin, G.A., Angioni, A., Barbanti-Brodano, G., and Negrini, M. (2002) Loss of methylation at chromosome 11p15.5 is common in human adult tumors. *Oncogene* **21(16),** 2564.
129. Horike, S., Mitsuya, K., Meguro, M., Kotobuki, N., Kashiwagi, A., Notsu, T., Schulz, T.C., Shirayoshi, Y., and Oshimura, M. (2000) Targeted disruption of the human LIT1 locus defines a putative imprinting control element playing an essential role in Beckwith-Wiedemann syndrome. *Hum. Mol. Genet*. **9(14),** 2075.
130. Murrell, A., Heeson, S., Cooper, W.N., Douglas, E., Apostolidou, S., Moore, G.E., Maher, E.R., and Reik, W. (2004) An association between variants in the IGF2 gene and Beckwith-Wiedemann syndrome: interaction between genotype and epigenotype. *Hum. Mol. Genet*. **13(2),** 247.
131. Murrell, A., Heeson, S., and Reik, W. (2004) Interaction between differentially methylated regions partitions the imprinted genes Igf2 and H19 into parent-specific chromatin loops. *Nat. Genet*. **36(8),** 889.
132. Chen, C.L., Ip, S.M., Cheng, D., Wong, L.C., and Ngan, H.Y. (2000) Loss of imprinting of the IGF-II and H19 genes in epithelial ovarian cancer. *Clin. Cancer Res*. **6(2),** 474.
133. Kim, K.S. and Lee, Y.I. (1997) Biallelic expression of the H19 and IGF2 genes in hepatocellular carcinoma. *Cancer Lett*. **119(2),** 143.
134. Sullivan, M.J., Taniguchi, T., Jhee, A., Kerr, N., and Reeve, A.E. (1999) Relaxation of IGF2 imprinting in Wilms tumours associated with specific changes in IGF2 methylation. *Oncogene* **18(52),** 7527.
135. Hofmann, W.K., Takeuchi, S., Frantzen, M.A., Hoelzer, D., and Koeffler, H.P. (2002) Loss of genomic imprinting of insulin-like growth factor 2 is strongly associated with cellular proliferation in normal hematopoietic cells. *Exp. Hematol*. **30(4),** 318.
136. Judson, H., van Roy, N., Strain, L., Vandesompele, J., Van Gele, M., Speleman, F., and Bonthron, D.T. (2000) Structure and mutation analysis of the gene encoding DNA fragmentation factor 40 (caspase-activated nuclease), a candidate neuroblastoma tumour suppressor gene. Hum. Genet. **106(4),** 406.
137. Luo, R.Z., Fang, X., Marquez, R., Liu, S.Y., Mills, G.B., Liao, W.S., Yu, Y., and Bast, R.C. (2003) ARHI is a Ras-related small G-protein with a novel N-terminal extension that inhibits growth of ovarian and breast cancers. *Oncogene* **22(19),** 2897.
138. Luo, R.Z., Peng, H., Xu, F., Bao, J., Pang, Y., Pershad, R., Issa, J.P., Liao, W.S., Bast, R.C., Jr., and Yu, Y. (2001) Genomic structure and promoter characterization of an imprinted tumor suppressor gene ARHI. *Biochim. Biophys.*

*Acta* **1519(3),** 216.
139. Arima, T., Drewell, R.A., Oshimura, M., Wake, N., and Surani, M.A. (2000) A novel imprinted gene, HYMAI, is located within an imprinted domain on human chromosome 6 containing ZAC. *Genomics* **67(3),** 248.
140. Abdollahi, A., Pisarcik, D., Roberts, D., Weinstein, J., Cairns, P., and Hamilton, T.C. (2003) LOT1 (PLAGL1/ZAC1), the candidate tumor suppressor gene at chromosome 6q24-25, is epigenetically regulated in cancer. *J. Biol. Chem.* **278(8),** 6041.
141. Kamikihara, T., Arima, T., Kato, K., Matsuda, T., Kato, H., Douchi, T., Nagata, Y., Nakao, M., and Wake, N. (2005) Epigenetic silencing of the imprinted gene ZAC by DNA methylation is an early event in the progression of human ovarian cancer. *Int. J. Cancer* **115(5),** 690.
142. Tsou, A.P., Chuang, Y.C., Su, J.Y., Yang, C.W., Liao, Y.L., Liu, W.K., Chiu, J.H., and Chou, C.K. (2003) Overexpression of a novel imprinted gene, PEG10, in human hepatocellular carcinoma and in regenerating mouse livers. *J. Biomed. Sci*. **10(6 Pt 1),** 625.
143. Okabe, H., Satoh, S., Furukawa, Y., Kato, T., Hasegawa, S., Nakajima, Y., Yamaoka, Y., and Nakamura, Y. (2003) Involvement of PEG10 in human hepatocellular carcinogenesis through interaction with SIAH1. *Cancer Res*. **63(12),** 3043.
144. Kayashima, T., Yamasaki, K., Yamada, T., Sakai, H., Miwa, N., Ohta, T., Yoshiura, K., Matsumoto, N., Nakane, Y., Kanetake, H., Ishino, F., Niikawa, N., and Kishino, T. (2003) The novel imprinted carboxypeptidase A4 gene ( CPA4) in the 7q32 imprinting domain. *Hum. Genet*. **112(3),** 220.
145. Nakanishi, H., Suda, T., Katoh, M., Watanabe, A., Igishi, T., Kodani, M., Matsumoto, S., Nakamoto, M., Shigeoka, Y., Okabe, T., Oshimura, M., and Shimizu, E. (2004) Loss of imprinting of PEG1/MEST in lung cancer cell lines. *Oncol. Rep*. **12(6),** 1273.
146. Pedersen, I.S., Dervan, P., McGoldrick, A., Harrison, M., Ponchel, F., Speirs, V., Isaacs, J.D., Gorey, T., and McCann, A. (2002) Promoter switch: a novel mechanism causing biallelic PEG1/MEST expression in invasive breast cancer. *Hum. Mol. Genet*. **11(12),** 1449.
147. Kohda, M., Hoshiya, H., Katoh, M., Tanaka, I., Masuda, R., Takemura, T., Fujiwara, M., and Oshimura, M. (2001) Frequent loss of imprinting of IGF2 and MEST in lung adenocarcinoma. *Mol. Carcinog*. **31(4),** 184.
148. Pedersen, I.S., Dervan, P.A., Broderick, D., Harrison, M., Miller, N., Delany, E., O'Shea, D., Costello, P., McGoldrick, A., Keating, G., Tobin, B., Gorey, T., and McCann, A. (1999) Frequent loss of imprinting of PEG1/MEST in invasive breast cancer. *Cancer Res*. **59(21),** 5449.
149. Tanaka, K., Shiota, G., Meguro, M., Mitsuya, K., Oshimura, M., and Kawasaki, H. (2001) Loss of imprinting of long QT intronic transcript 1 in colorectal cancer. *Oncology* **60(3),** 268.
150. Hartmann, W., Waha, A., Koch, A., Goodyer, C.G., Albrecht, S., von Schweinitz, D., and Pietsch, T. (2000) p57(KIP2) is not mutated in hepatoblastoma but shows increased transcriptional activity in a comparative analysis of the three imprinted genes p57(KIP2), IGF2, and H19. *Am. J. Pathol*. **157(4),** 1393.
151. Lai, S., Goepfert, H., Gillenwater, A.M., Luna, M.A., and El-Naggar, A.K. (2000) Loss of imprinting and genetic alterations of the cyclin-dependent kinase inhibitor p57KIP2 gene in head and neck squamous cell carcinoma. *Clin. Cancer Res*. **6(8),** 3172.
152. Anderson, J., Gordon, A., McManus, A., Shipley, J., and Pritchard-Jones, K. (1999) Disruption of imprinted genes at chromosome region 11p15.5 in paediatric rhabdomyosarcoma. *Neoplasia* **1(4),** 340.
153. Ito, Y., Yoshida, H., Nakano, K., Kobayashi, K., Yokozawa, T., Hirai, K., Matsuzuka, F., Matsuura, N., Kuma, K., and Miyauchi, A. (2002) Expression of p57/Kip2 protein in normal and neoplastic thyroid tissues. *Int. J. Mol. Med*. **9(4),** 373.
154. Ito, Y., Takeda, T., Wakasa, K., Tsujimoto, M., and Matsuura, N. (2001) Expression of p57/Kip2 protein in pancreatic adenocarcinoma. *Pancreas* **23(3),** 246.
155. Ito, Y., Takeda, T., Sakon, M., Tsujimoto, M., Monden, M., and Matsuura, N. (2001) Expression of p57/Kip2 protein in hepatocellular carcinoma. *Oncology* **61(3),** 221.
156. Dallosso, A.R., Hancock, A.L., Brown, K.W., Williams, A.C., Jackson, S., and Malik, K. (2004) Genomic imprinting at the WT1 gene involves a novel coding transcript (AWT1) that shows deregulation in Wilms' tumours. *Hum. Mol. Genet*. **13(4),** 405.
157. Baysal, B.E. (2004) Genomic imprinting and environment in hereditary paraganglioma. *Am. J. Med. Genet. C Semin. Med. Genet*. **129(1),** 85.
158. Astuti, D., Morris, M., Krona, C., Abel, F., Gentle, D., Martinsson, T., Kogner, P., Neumann, H.P., Voutilainen, R., Eng, C., Rustin, P., Latif, F., and Maher, E.R. (2004) Investigation of the role of SDHB inactivation in sporadic phaeochromocytoma and neuroblastoma. *Br. J. Cancer* **91(10),** 1835.
159. Astuti, D., Latif, F., Wagner, K., Gentle, D., Cooper, W.N., Catchpoole, D., Grundy, R., Ferguson-Smith, A.C., and Maher, E.R. (2005) Epigenetic alteration at the DLK1-GTL2 imprinted domain in human neoplasia: analysis of neuroblastoma, phaeochromocytoma and Wilms' tumour. *Br. J. Cancer*. **92(8),** 1574.
160. Maegawa, S., Itaba, N., Otsuka, S., Kamitani, H., Watanabe, T., Tahimic, C.G., Nanba, E., and Oshimura, M. (2004) Coordinate downregulation of a novel imprinted transcript ITUP1 with PEG3 in glioma cell lines. *DNA Res*. **11(1),** 37.
161. Maegawa, S., Yoshioka, H., Itaba, N., Kubota, N., Nishihara, S., Shirayoshi, Y., Nanba, E., and Oshimura, M. (2001) Epigenetic silencing of PEG3 gene expression in human glioma cell lines. *Mol. Carcinog*. **31(1),** 1.

162. Kuerbitz, S.J., Pahys, J., Wilson, A., Compitello, N., and Gray, T.A. (2002) Hypermethylation of the imprinted NNAT locus occurs frequently in pediatric acute leukemia. *Carcinogenesis* **23(4),** 559.
163. Li, J., Bench, A.J., Vassiliou, G.S., Fourouclas, N., Ferguson-Smith, A.C., and Green, A.R. (2004) Imprinting of the human L3MBTL gene, a polycomb family member located in a region of chromosome 20 deleted in human myeloid malignancies. *Proc. Natl. Acad. Sci. U. S. A.* **101(19),** 7341.
164. Farrell, W.E. (2005) Epigenetic mechanisms of tumorigenesis. *Horm. Metab. Res*. **37(6),** 361.
165. Collins, M.T., Sarlis, N.J., Merino, M.J., Monroe, J., Crawford, S.E., Krakoff, J.A., Guthrie, L.C., Bonat, S., Robey, P.G., and Shenker, A. (2003) Thyroid carcinoma in the McCune-Albright syndrome: contributory role of activating Gs alpha mutations. *J. Clin. Endocrinol. Metab.* **88(9),** 4413.
166. Jackson-Grusby, L., Beard, C., Possemato, R., Tudor, M., Fambrough, D., Csankovszki, G., Dausman, J., Lee, P., Wilson, C., Lander, E., and Jaenisch, R. (2001) Loss of genomic methylation causes p53-dependent apoptosis and epigenetic deregulation. *Nat. Genet.* **27(1),** 31.
167. Caspary, T., Cleary, M.A., Baker, C.C., Guan, X.J., and Tilghman, S.M. (1998) Multiple mechanisms regulate imprinting of the mouse distal chromosome 7 gene cluster. *Mol. Cell Biol.* **18(6),** 3466.
168. Okano, M., Bell, D.W., Haber, D.A., and Li, E. (1999) DNA methyltransferases Dnmt3a and Dnmt3b are essential for de novo methylation and mammalian development. *Cell* **99(3),** 247.
169. Jeffery, L. and Nakielny, S. (2004) Components of the DNA methylation system of chromatin control are RNA-binding proteins. *J. Biol. Chem*. **279(47),** 49479.
170. Jiang, C.L., Jin, S.G., and Pfeifer, G.P. (2004) MBD3L1 is a transcriptional repressor that interacts with methyl-CpG-binding protein 2 (MBD2) and components of the NuRD complex. *J. Biol. Chem*. **279(50),** 52456.
171. Kantor, B., Makedonski, K., Shemer, R., and Razin, A. (2003) Expression and localization of components of the histone deacetylases multiprotein repressory complexes in the mouse preimplantation embryo. *Gene Expr. Patterns* **3(6),** 697.
172. Nan, X. and Bird, A. (2001) The biological functions of the methyl-CpG-binding protein MeCP2 and its implication in Rett syndrome. *Brain Dev*. **23(Suppl 1),** S32.
173. Makedonski, K., Abuhatzira, L., Kaufman, Y., Razin, A., and Shemer, R. (2005) MeCP2 deficiency in Rett syndrome causes epigenetic aberrations at the PWS/AS imprinting center that affects UBE3A expression. *Hum. Mol. Genet*. **14(8),** 1049.
174. Horike, S., Cai, S., Miyano, M., Cheng, J.F., and Kohwi-Shigematsu, T. (2005) Loss of silent-chromatin looping and impaired imprinting of DLX5 in Rett syndrome. *Nat. Genet*. **37(1),** 31.
175. Filion, G.J., Zhenilo, S., Salozhin, S., Yamada, D., Prokhortchouk, E., and Defossez, P.A. (2006) A family of human zinc finger proteins that bind methylated DNA and repress transcription. *Mol. Cell. Biol*. **26(1),** 169.
176. Defossez, P.A., Kelly, K.F., Filion, G.J., Perez-Torrado, R., Magdinier, F., Menoni, H., Nordgaard, C.L., Daniel, J.M., and Gilson, E. (2005) The human enhancer blocker CTC-binding factor interacts with the transcription factor Kaiso. *J. Biol. Chem*. **280(52),** 43017.
177. Morgan, H.D., Dean, W., Coker, H.A., Reik, W., and Petersen-Mahrt, S.K. (2004) Activation-induced cytidine deaminase deaminates 5-methylcytosine in DNA and is expressed in pluripotent tissues: implications for epigenetic reprogramming. *J. Biol. Chem*. **279(50),** 52353.
178. Santos-Rosa, H. and Caldas, C. (2005) Chromatin modifier enzymes, the histone code and cancer. *Eur. J. Cancer* **41(16),** 2381.
179. Wang, H., Huang, Z.Q., Xia, L., Feng, Q., Erdjument-Bromage, H., Strahl, B.D., Briggs, S.D., Allis, C.D., Wong, J., Tempst, P., and Zhang, Y. (2001) Methylation of histone H4 at arginine 3 facilitating transcriptional activation by nuclear hormone receptor. *Science* **293(5531),** 853.
180. Nishioka, K., Chuikov, S., Sarma, K., Erdjument-Bromage, H., Allis, C.D., Tempst, P., and Reinberg, D. (2002) Set9, a novel histone H3 methyltransferase that facilitates transcription by precluding histone tail modifications required for heterochromatin formation. *Genes Dev*. **16(4),** 479.
181. Peters, A.H., O'Carroll, D., Scherthan, H., Mechtler, K., Sauer, S., Schofer, C., Weipoltshammer, K., Pagani, M., Lachner, M., Kohlmaier, A., Opravil, S., Doyle, M., Sibilia, M., and Jenuwein, T. (2001) Loss of the Suv39h histone methyltransferases impairs mammalian heterochromatin and genome stability. *Cell* **107(3),** 323.
182. Schotta, G., Lachner, M., Sarma, K., Ebert, A., Sengupta, R., Reuter, G., Reinberg, D., and Jenuwein, T. (2004) A silencing pathway to induce H3-K9 and H4-K20 trimethylation at constitutive heterochromatin. *Genes Dev*. **18(11),** 1251.
183. Dou, Y., Milne, T.A., Tackett, A.J., Smith, E.R., Fukuda, A., Wysocka, J., Allis, C.D., Chait, B.T., Hess, J.L., and Roeder, R.G. (2005) Physical association and coordinate function of the H3 K4 methyltransferase MLL1 and the H4 K16 acetyltransferase MOF. *Cell* **121(6),** 873.
184. Cao, R. and Zhang, Y. (2004) The functions of E(Z)/EZH2-mediated methylation of lysine 27 in histone H3. *Curr. Opin. Genet. Dev.* **14(2),** 155.
185. Shi, Y., Lan, F., Matson, C., Mulligan, P., Whetstine, J.R., Cole, P.A., and Casero, R.A. (2004) Histone demethylation mediated by the nuclear amine oxidase homolog LSD1. *Cell* **119(7),** 941.
186. Shi, Y.J., Matson, C., Lan, F., Iwase, S., Baba, T., and Shi, Y. (2005) Regulation of LSD1 histone demethylase activity by its associated factors. *Mol. Cell* **19(6),** 857.
187. Cuthbert, G.L., Daujat, S., Snowden, A.W., Erdjument-Bromage, H., Hagiwara, T., Yamada, M., Schneider, R.,

Gregory, P.D., Tempst, P., Bannister, A.J., and Kouzarides, T. (2004) Histone deimination antagonizes arginine methylation. *Cell* **118(5),** 545.
188. Klenova, E.M., Nicolas, R.H., Paterson, H.F., Carne, A.F., Heath, C.M., Goodwin, G.H., Neiman, P.E., and Lobanenkov, V.V. (1993) CTCF, a conserved nuclear factor required for optimal transcriptional activity of the chicken c-myc gene, is an 11-Zn-finger protein differentially expressed in multiple forms. *Mol. Cell Biol.* **13(12),** 7612.
189. Klenova, E.M., Morse, H.C., 3rd, Ohlsson, R., and Lobanenkov, V.V. (2002) The novel BORIS + CTCF gene family is uniquely involved in the epigenetics of normal biology and cancer. *Semin. Cancer Biol.* **12(5),** 399.
190. Du, M., Beatty, L.G., Zhou, W., Lew, J., Schoenherr, C., Weksberg, R., and Sadowski, P.D. (2003) Insulator and silencer sequences in the imprinted region of human chromosome 11p15.5. *Hum. Mol. Genet.* **12(15),** 1927.
191. Yoon, B., Herman, H., Hu, B., Park, Y.J., Lindroth, A., Bell, A., West, A.G., Chang, Y., Stablewski, A., Piel, J.C., Loukinov, D.I., Lobanenkov, V.V., and Soloway, P.D. (2005) Rasgrf1 imprinting is regulated by a CTCF-dependent methylation-sensitive enhancer blocker. *Mol. Cell Biol.* **25(24),** 11184.
192. Rosa, A.L., Wu, Y.Q., Kwabi-Addo, B., Coveler, K.J., Reid Sutton, V., and Shaffer, L.G. (2005) Allele-specific methylation of a functional CTCF binding site upstream of MEG3 in the human imprinted domain of 14q32. *Chromosome Res.* **13(8),** 809.
193. Dennis, K., Fan, T., Geiman, T., Yan, Q., and Muegge, K. (2001) Lsh, a member of the SNF2 family, is required for genome-wide methylation. *Genes Dev.* **15(22),** 2940.
194. Fan, T., Yan, Q., Huang, J., Austin, S., Cho, E., Ferris, D., and Muegge, K. (2003) Lsh-deficient murine embryonal fibroblasts show reduced proliferation with signs of abnormal mitosis. *Cancer Res.* **63(15),** 4677.
195. Fan, T., Hagan, J.P., Kozlov, S.V., Stewart, C.L., and Muegge, K. (2005) Lsh controls silencing of the imprinted Cdkn1c gene. *Development* **132(4),** 635.
196. Bannister, A.J., Zegerman, P., Partridge, J.F., Miska, E.A., Thomas, J.O., Allshire, R.C., and Kouzarides, T. (2001) Selective recognition of methylated lysine 9 on histone H3 by the HP1 chromo domain. *Nature* **410(6824),** 120.
197. Lachner, M., O'Carroll, D., Rea, S., Mechtler, K., and Jenuwein, T. (2001) Methylation of histone H3 lysine 9 creates a binding site for HP1 proteins. *Nature* **410(6824),** 116.
198. Hinkins, M., Huntriss, J., Miller, D., and Picton, H.M. (2005) Expression of Polycomb-group genes in human ovarian follicles, oocytes and preimplantation embryos. *Reproduction* **130(6),** 883.
199. Plath, K., Fang, J., Mlynarczyk-Evans, S.K., Cao, R., Worringer, K.A., Wang, H., de la Cruz, C.C., Otte, A.P., Panning, B., and Zhang, Y. (2003) Role of histone H3 lysine 27 methylation in X inactivation. *Science* **300(5616),** 131.
200. Wang, J., Mager, J., Chen, Y., Schneider, E., Cross, J.C., Nagy, A., and Magnuson, T. (2001) Imprinted X inactivation maintained by a mouse Polycomb group gene. *Nat. Genet.* **28(4),** 371.
201. Mager, J., Montgomery, N.D., de Villena, F.P., and Magnuson, T. (2003) Genome imprinting regulated by the mouse Polycomb group protein Eed. *Nat. Genet.* **33(4),** 502.
202. Delaval, K. and Feil, R. (2004) Epigenetic regulation of mammalian genomic imprinting. *Curr. Opin. Genet. Dev.* **14(2),** 188.
203. Kim, J., Kollhoff, A., Bergmann, A., and Stubbs, L. (2003) Methylation-sensitive binding of transcription factor YY1 to an insulator sequence within the paternally expressed imprinted gene, Peg3. *Hum. Mol. Genet.* **12(3),** 233.
204. Murdoch, S., Djuric, U., Mazhar, B., Seoud, M., Khan, R., Kuick, R., Bagga, R., Kircheisen, R., Ao, A., Ratti, B., Hanash, S., Rouleau, G.A., and Slim. R. (2006) Mutations in NALP7 cause recurrent hydatidiform moles and reproductive wastage in humans. *Nat Genet.* **38(3)**, 300-302.
205. Gallagher, E., Mc Goldrick, A., Chung, W.Y., Mc Cormack, O., Harrison, M., Kerin, M., Dervan, P.A., and Mc Cann, A. (2006) Gain of imprinting of SLC22A18 sense and antisense transcripts in human breast cancer. *Genomics.* Apr 17.
206. Yu, Y., Xu, F., Peng, H., Fang, X., Zhao, S., Li, Y., Cuevas, B., Kuo, W.L., Gray, J.W., Siciliano, M., Mills, G.B., and Bast, R.C. Jr. (1999) NOEY2 (ARHI), an imprinted putative tumor suppressor gene in ovarian and breast carcinomas. *Proc Natl Acad Sci U S A.* **96(1),** 214-219.
207. Kurukuti, S., Tiwari, V., Tavosidana, G., Pugacheva, E., Murrell, A., Zhao, Z., Lobanenkov, V., Reik, W., and Ohlsson R. (2006) CTCF binding at the H19 ICR mediates maternally-inherited higher order chromatin conformation and regional epigenotype to restrict enhancer access to Igf2. *PNAS* (manuscript in revision)
